# Slik maintains tissue homeostasis by preventing JNK-mediated apoptosis

**DOI:** 10.1186/s13008-023-00097-4

**Published:** 2023-10-04

**Authors:** Chenglin Li, Xiaojie Zhu, Xinyue Sun, Xiaowei Guo, Wenzhe Li, Ping Chen, Yulii V. Shidlovskii, Qian Zhou, Lei Xue

**Affiliations:** 1 The First Rehabilitation Hospital of Shanghai, Shanghai Key Laboratory of Signaling and Diseases Research, School of Life Science and Technology, Tongji University, Shanghai, China; 2 The Key Laboratory of Model Animals and Stem Cell Biology in Hunan Province, School of Medicine, Hunan Normal University, Changsha, Hunan China; 3 Department of Gene Expression Regulation in Development, Institute of Gene Biology, Russian Academy of Sciences, Moscow, Russia; 4 Department of Biology and General Genetics, Sechenov University, 8, bldg. 2 Trubetskaya St, Moscow, 119048 Russia; 5 Guangdong Provincial Key Laboratory of Tumor Interventional Diagnosis and Treatment, Zhuhai Precision Medical Center, Zhuhai People’s Hospital, Zhuhai Hospital Affiliated with Jinan University, Zhuhai, Guangdong China

**Keywords:** *Slik*, JNK, *Drosophila*, Apoptosis, Homeostasis

## Abstract

**Background:**

The c-Jun N-terminal kinase (JNK) pathway is an evolutionarily conserved regulator of cell death, which is essential for coordinating tissue homeostasis. In this study, we have characterized the *Drosophila* Ste20-like kinase Slik as a novel modulator of JNK pathway-mediated apoptotic cell death.

**Results:**

First, ectopic JNK signaling-triggered cell death is enhanced by *slik* depletion whereas suppressed by Slik overexpression. Second, loss of *slik* activates JNK signaling, which results in enhanced apoptosis and impaired tissue homeostasis. In addition, genetic epistasis analysis suggests that Slik acts upstream of or in parallel to Hep to regulate JNK-mediated apoptotic cell death. Moreover, Slik is necessary and sufficient for preventing physiologic JNK signaling-mediated cell death in development. Furthermore, introduction of STK10, the human ortholog of Slik, into *Drosophila* restores *slik* depletion-induced cell death and compromised tissue homeostasis. Lastly, knockdown of *STK10* in human cancer cells also leads to JNK activation, which is cancelled by expression of Slik.

**Conclusions:**

This study has uncovered an evolutionarily conserved role of Slik/STK10 in blocking JNK signaling, which is required for cell death inhibition and tissue homeostasis maintenance in development.

**Supplementary Information:**

The online version contains supplementary material available at 10.1186/s13008-023-00097-4.

## Background

Tissue homeostasis depends on highly coordinated cellular processes, including cell regeneration, proliferation, differentiation, and apoptosis [1–5]. The evolutionarily conserved c-Jun N-terminal kinase (JNK) signaling is one of the key pathways that govern a wide range of biological processes including stress response, cell proliferation and apoptosis, which facilitates the maintenance of tissue homeostasis in development [6–9]. JNK signaling is initiated by various intrinsic and extrinsic signals, and is mediated by a mitogen-activated protein kinase (MAPK) cascade [10, 11]. In *Drosophila*, JNK signaling is activated by the tumor necrosis factor (TNF) ortholog Eiger (Egr), which binds to its receptor Wengen (Wgn) or Grindelwald (Grnd) that recruits the TNF receptor-associated factor 2 (dTRAF2) [12–14] to trigger the conserved MAP kinase cascade including dTAK1 (JNKK kinase), Hemipterous (Hep, JNK kinase) and Basket (Bsk, *Drosophila* JNK) [15–17]. Phosphorylated Bsk translocates into the nucleus to phosphorylate and activate the transcription factor complex AP-1, which upregulates the transcription of target genes [11]. The *puckered* (*puc*) gene, which encodes a JNK phosphatase, is a transcriptional target of JNK signaling, and thereby regulates JNK activity in a negative feedback manner [18, 19].

The Sterile-20 (Ste20) kinases have been implicated in numerous physiological processes including cell proliferation and survival [20], cytoskeletal dynamics [21, 22], and ion transport [23]. The Ste20-kinase family consists of a heterogeneous group of Ser/Thr kinases including two large families (PAK- and GCK-like) and ten divergent subfamilies [21, 24]. *Drosophila* Slik and its mammalian ortholog STK10 belong to the GCK-V subfamily [25], which has emerged as an evolutionarily conserved modulator of ezrin/radixin/moesin (ERM) family proteins [26, 27]. In *Drosophila*, Moesin and Merlin, two FERM domain proteins, are the best known substrates of Slik [28]. While Slik-mediated phosphorylation activates Moesin to stabilize microtubule organization and epithelial integrity [29, 30], phosphorylation by Slik inactivates Merlin to adjust cell proliferation [31, 32]. Most of the physiological activities are governed by signaling pathways, though Slik is known to participate in a variety of cellular processes, the connection between Slik and potential signaling pathway remains poorly understood.

*Drosophila* is one of the best model organisms to study genetics, with many components of signal pathways being identified in fruit flies. We have previously performed a genetic screen with ectopic JNK-triggered cell death, and have characterized multiple components and regulators of the JNK pathway in *Drosophila* [33, 34]. In the current study, we report that Slik is required for maintaining tissue homeostasis by preventing JNK-mediated apoptotic cell death in development. Loss of *slik* leads to JNK activation and triggers JNK-mediated cell death. Genetic epistasis analysis suggests that Slik acts upstream of or in parallel to Hep to suppress JNK pathway. Finally, the human ortholog STK10 has retained Slik functions to modulate JNK activity when introduced into *Drosophila* or in human cells. Taken together, this study unravels a crucial role of Slik in maintaining tissue homeostasis and organ development by preventing JNK-mediated apoptotic cell death.

## Results

### Slik inhibits ectopic JNK-induced cell death in development

**Fig. 1:**
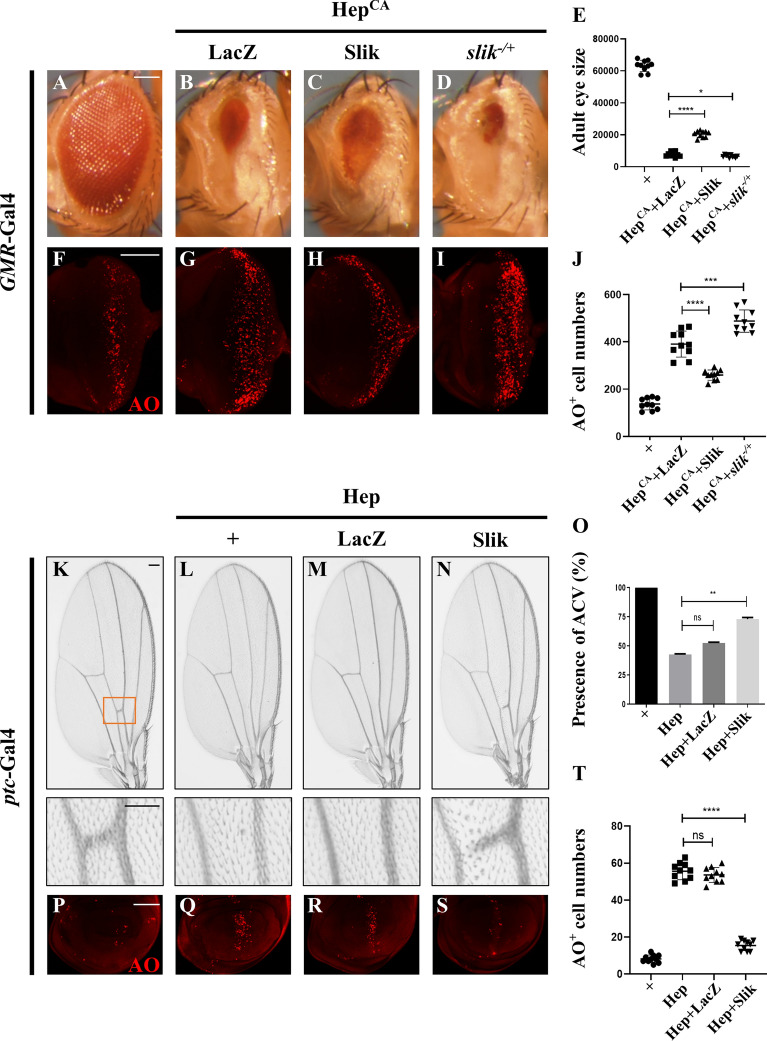
Slik inhibits ectopic Hep-induced cell death. Light micrographs of *Drosophila* adult eyes **A**–**D** and fluorescence micrographs of third instar larval eye discs **F**–**I** are shown. Compared with the *GMR*-Gal4 controls (**A**, **F**, *GMR* > Hep^CA^-induced small eye phenotype **B** and cell death in eye discs **G** were partially inhibited by overexpression of Slik **C**, **H**, whereas enhanced in heterozygous *slik* mutants **D**, **I**. Statistical analysis of the adult eye sizes (**E**, n = 10 for each genotype) and AO-positive cell numbers in eye discs (**J**, n = 10 for each genotype) are shown. Light micrographs of *Drosophila* adult wings **K**–**N** and fluorescence micrographs of third instar larval wing discs **P**–**S** are shown. Compared with the *ptc*-Gal4 controls **K**, **P**, ectopic expression of Hep driven by *ptc*-Gal4 generated a loss-of-ACV phenotype in adults **L** and induced cell death in larval wing discs (**Q**), which were strongly blocked by expressing Slik (**N, S**), but not LacZ (**M**, **R**). The lower panels show high magnification view of the boxed areas in upper panels **K**–**N**). Quantification of the presence of ACV in adult wings (**O**, n = 15 for each genotype) and cell death in wing discs (**T**, n = 10 for each genotype) are shown. One-way ANOVA was used to compute P-values, ****p < 0.0001, ***p < 0.001, **p < 0.01, *P < 0.05, n.s indicates not significant. See the supplementary material for detailed genotypes. Scale bar:100 μm in **A**–**D**, **F**–**I**, **K**–**N** (upper panels) and **P**–**S**, 50 μm in **K**–**N** (lower panels)

Given that JNK signaling is of vital importance in regulating cell death, to identify new factors that regulate JNK-mediated cell death, we performed a genetic screen for dominant modifiers of *GMR* > Egr-induced cell death in the *Drosophila* eyes using the Bloomington *Drosophila* Stock Center Deficiency kit [5, 35]. We found that three overlapping deficiencies, *Df(2R)BSC603*, *Df(2R)ED4065* and *Df(2R)ED4071*, enhanced the small eye phenotype caused by *GMR* > Egr, indicating a potential enhancer is located within the common region (Additional file 1: Fig. S1A–E). This was confirmed by subsequent experiments showing that either mutation or knockdown of *slik*, one of the genes in this region, significantly enhanced the *GMR* > Egr-induced small eye phenotype (Additional file 1^1^ Fig. S1F, G). Furthermore, ectopic expression of a constitutive active form of Hep (Hep^CA^) triggered strong cell death posterior to the morphogenetic furrow (MF) in the eye imaginal discs (Fig. 1F, G), indicated by Acridine Orange (AO) staining that detects dying cells [36], and produced a small eye phenotype in the adults (Fig. 1A, B). We found that *GMR* > Hep^CA^-induced cell death and small eye phenotype were enhanced in heterozygous *slik* mutants, but were significantly suppressed by overexpression of Slik (Fig. 1C–E, H–J).

To investigate whether Slik inhibits JNK-mediated cell death in other tissues, we turned to the developing wing. Compared with the controls (Fig. 1K, P), expression of Hep along the anterior/posterior (A/P) compartment border driven by *patched* (*ptc*)-Gal4 [37] resulted in increased cell death in the wing imaginal discs (Fig. 1Q), and generated a loss of anterior cross vein (ACV) phenotype in the adult wings (Fig. 1L). Both phenotypes were considerably suppressed by ectopic expression of Slik, but remained unaffected by that of LacZ (Fig. 1M–O, R–T). Collectively, these results indicate that Slik negatively regulates ectopic JNK-induced cell death during eye and wing development.

### Depletion of ***slik*** triggers apoptotic cell death in development

**Fig. 2:**
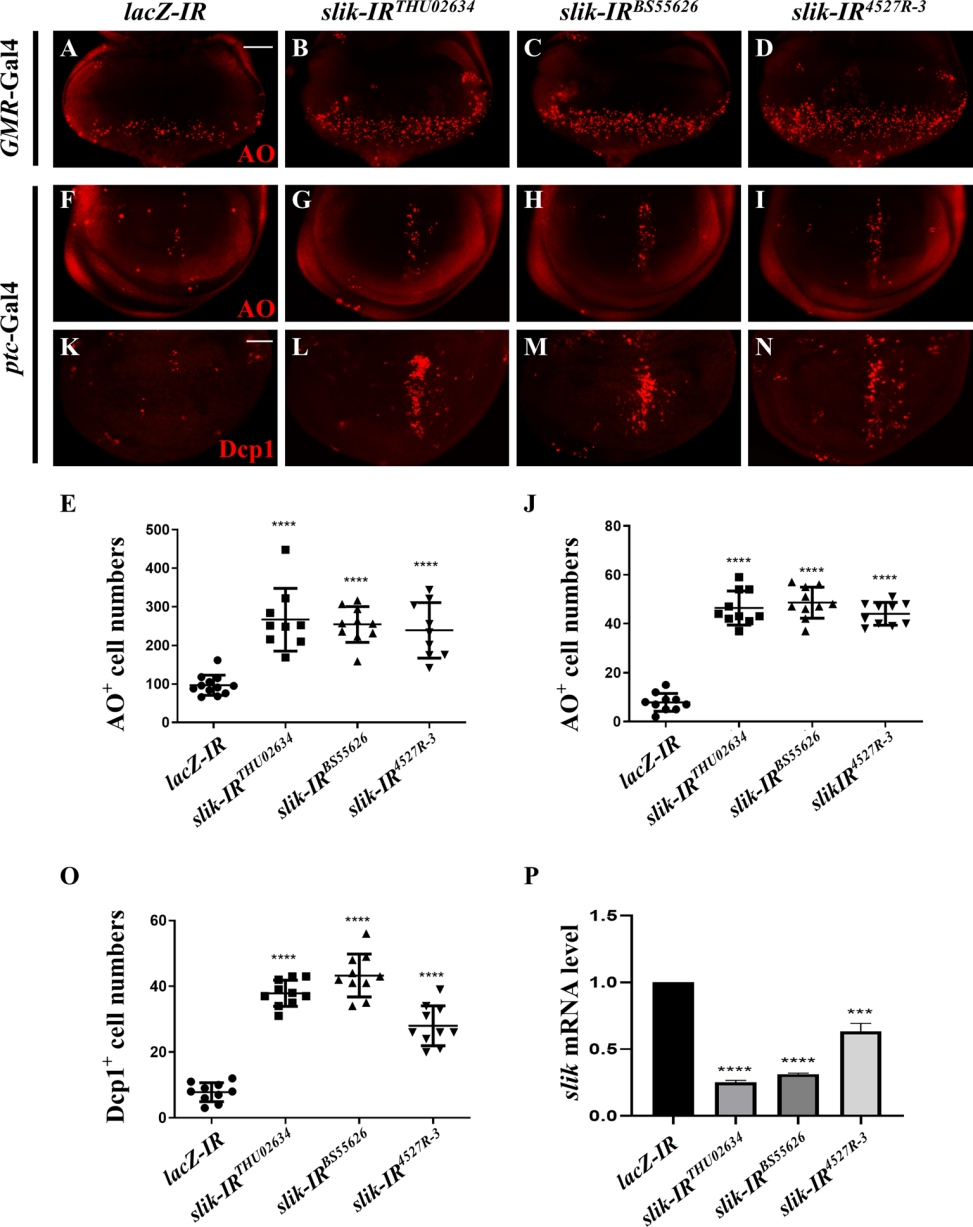
Depletion of *slik* triggers apoptotic cell death. Fluorescence micrographs of third instar larval eye discs **A**–**D** are shown. Compared with the *GMR*-Gal4 controls (**A**), downregulation of *slik* with three independent RNA interference (RNAi) triggered extensive cell death posterior to the MF (**B**–**D**). Fluorescence micrographs of third instar larval wing discs **F**–**I**, **K**–**N** are shown. Compared with the *ptc*-Gal4 controls (**F**, **K**), depletion of *slik* induced massive cell death (**G**–**I**) and apoptosis **L**–**N** in wing discs. Quantification of cell death in eye discs (**E**, n = 12; n = 9; n = 10; n = 9) and statistical analysis of AO positive cell number (**J**, n = 10 for each genotype) and cleaved Dcp-1 (cDcp-1) activity (**O**, n = 10 for each genotype) in wing discs are shown. **P** The knock-down efficacies of *slik* RNAi lines. Expression of three independent *slik* RNAi significantly reduces the level of *slik* mRNA, as measured by quantitative RT-PCR. One-way ANOVA with Bonferroni multiple-comparison test was used to compute P-values, ****P < 0.0001, ***P < 0.001. See the supplementary material for detailed genotypes. Scale bar: 50 μm

Given that Slik is an inhibitor of ectopic JNK-mediated cell death, we wonder whether *slik* is physiologically required for preventing cell death in development. To test this, we depleted *slik* with three independent RNA interference (RNAi), whose knockdown efficiencies were confirmed by the quantitative reverse transcription polymerase chain reaction (RT-qPCR) assay (Fig. 2P). Compared with the *GMR*-Gal4 or *ptc*-Gal4 controls, cell death was significantly increased upon *slik* knockdown in the corresponding areas of 3rd instar larval eye (Fig. 2A–E) or wing (Fig. 2F–J) discs, suggesting that endogenous Slik inhibits cell death in normal development.

Apoptosis is the major form of cell death in *Drosophila*, which is mediated by the cleavage and activation of caspases [38, 39]. To check whether Slik regulates apoptotic cell death, we utilized an anti-cleaved Dcp-1 antibody that specifically recognizes the activated form of the effector caspase Dcp-1 [40]. We found that knockdown of *slik* induced strong apoptosis in the wing discs (Fig. 2K–O). Consistently, *slik* depletion-induced cell death was efficiently blocked by expressing P35, a viral caspase inhibitor [41], but not a *lacZ RNAi* (Additional file 1: Fig. S2). Taken together, these data suggest that *slik* is physiologically required for preventing apoptotic cell death in normal development.

### Depletion of ***slik*** promotes JNK pathway activation

**Fig. 3:**
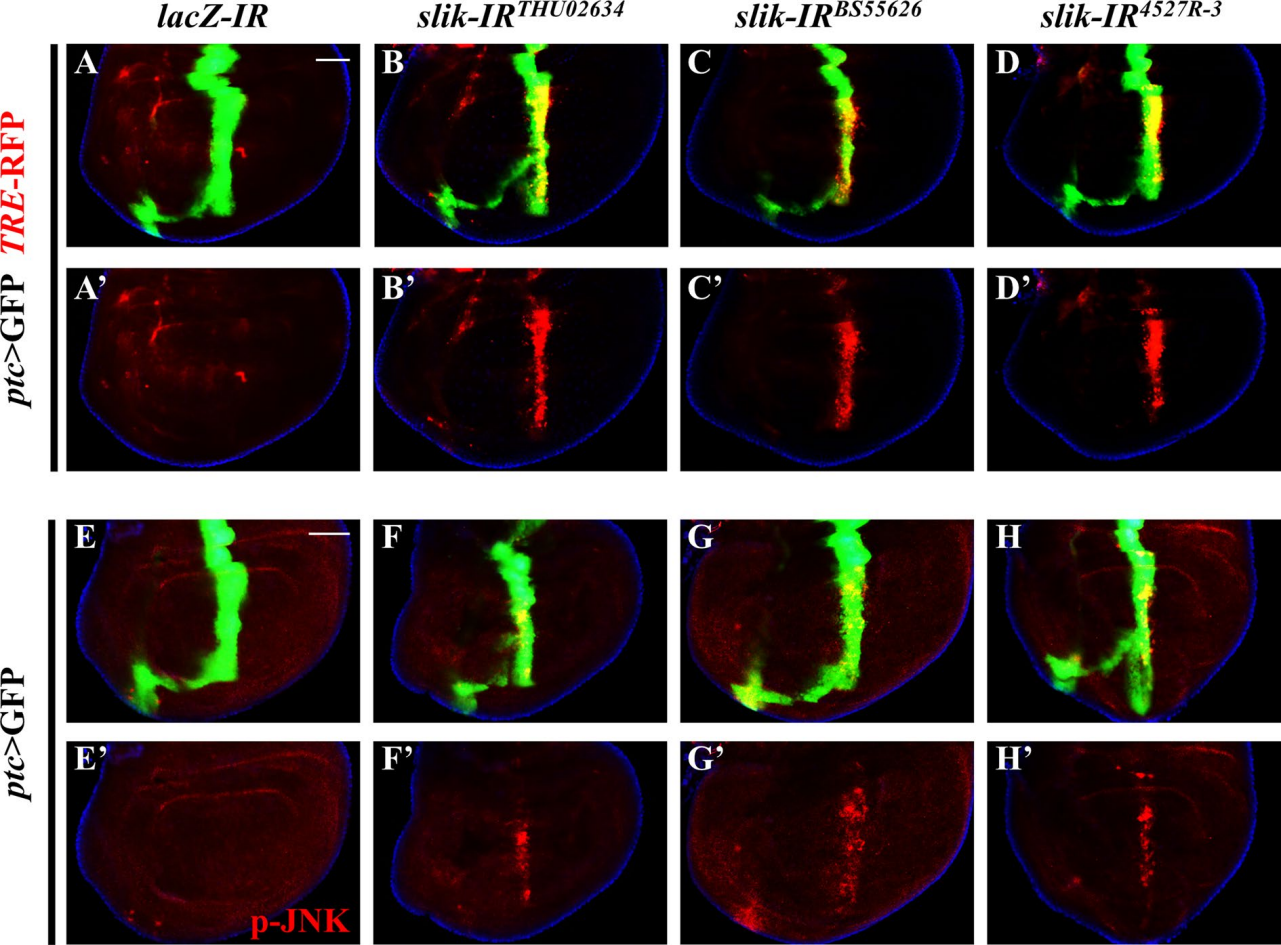
Loss of *slik* promotes JNK pathway activation in vivo. Merged fluorescence micrographs of *Drosophila* third instar larval wing discs are shown **A**–**H**. The red channels detected only RFP **A**’–**D**’ or pJNK signal **E**’–**H**’. Compared with the controls **A**, **E**, expressing three independent *slik* RNAi notably elevated the expression of *TRE*-RFP **B**–**D** and JNK phosphorylation (F-H) along the A/P compartment boundary. See the supplementary material for detailed genotype. Scale bar: 50 μm

As JNK signaling plays significant roles in regulating apoptotic cell death [42–45], *slik* depletion-triggered developmental apoptosis may depend on JNK pathway activation. In agreement with this assumption, knockdown of *slik*, but not *lacZ*, by *ptc-*Gal4 resulted in upregulated expression of *TRE*-RFP (Fig. 3A–D, A’-D’), a general reporter of JNK signaling [46, 47]. Moreover, JNK phosphorylation, detected by an antibody specific to the phosphorylated JNK (p-JNK) [5, 48], was considerably elevated upon *slik* depletion (Fig. 3E–H, E’–H’). Together, these data suggest that loss of *slik* potentiates JNK signaling in development.

### JNK signaling is required for loss of ***slik***-induced apoptotic cell death

**Fig. 4:**
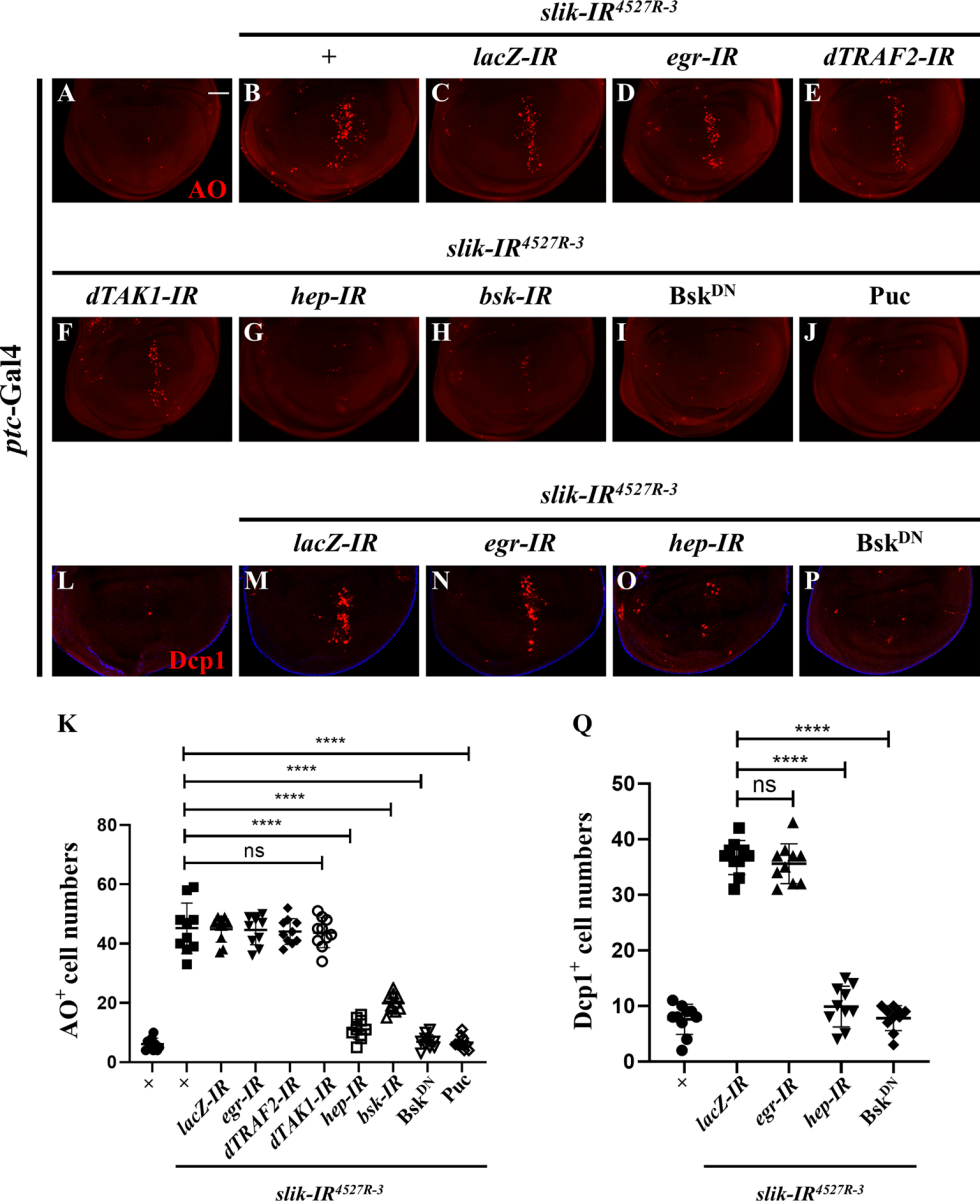
Slik acts upstream of or in parallel to Hep to adjust JNK-dependent apoptotic cell death. Fluorescence micrographs of third instar larval wing discs are shown (**A**–**J**). Compared with the control (**A**), *RNAi*-mediated down-regulation of *slik* along the A/P compartment boundary by *ptc*-Gal4 induced obvious cell death (**B**), which was suppressed by depletion of *hep* (**G**) or *bsk* (**H**), or expressing a dominant-negative form of Bsk (**I**) or Puc (**J**), but remained unaffected by expressing RNAi of *lacZ* (**C**), *egr* (**D**), *dTRAF2* (**E**) or *dTAK1* (**F**). Merged fluorescence micrographs of third instar larval wing discs are shown (**L**–**P**). Compared with the control (**L**), loss of *slik*-induced apoptosis **M** was blocked by expressing *hep* RNAi **O** or Bsk^DN^ (**P**), but not by *egr* RNAi (**N**). Statistical analysis of the AO positive cell numbers (**K**, n = 10 for each genotype) and cleaved Dcp-1 (cDcp-1) activity (**Q**, n = 10 for each genotype) in wing discs are shown. One-way ANOVA with Bonferroni multiple-comparison test was used to compute P-values, ****P < 0.0001, n.s indicates not significant. See the supplementary material for detailed genotypes. Scale bar: 50 μm

Given that *slik* depletion activates JNK and apoptosis, we hypothesized that loss of *slik* might promotes cell death through activating JNK signaling. In agreement with this speculation, we found that *ptc*-Gal4 (Fig. 4A) driven *slik* depletion-caused cell death (Fig. 4B) was soundly suppressed by knocking down *bsk*, which encodes the *Drosophila* JNK, or expressing a dominant negative form of Bsk (Bsk^DN^) or Puc, an inhibitor of JNK [18, 19] (Fig. 4H–K). Consistently, loss of *slik*-induced apoptosis, visualized by anti-cleaved Dcp-1 (cDcp-1) antibody staining, was remarkably inhibited by Bsk^DN^ (Fig. 4L, M, P, Q). In conclusion, these results suggest that *slik* depletion triggers JNK-dependent apoptotic cell death.

### Slik functions upstream of or in parallel to Hep

To understand how Slik regulates JNK signaling-mediated cell death, we performed genetic epistasis analysis between Slik and core components of the Egr-JNK pathway. We found that *slik* knockdown-induced cell death was significantly suppressed by depletion of *hep*, but not by that of *egr*, *dTRAF2*, *dTAK1* or *lacZ* serving as a negative control (Fig. 4A–G, K). Consistently, loss of *slik*-induced Dcp-1 activation was abolished by expression of *hep-IR* or Bsk^DN^, but remained unaffected upon depletion of *egr* (Fig. 4L–Q). Taken together, these results suggest that Slik acts upstream of or in parallel to Hep to regulate JNK-mediated apoptotic cell death.

### Slik modulates JNK-dependent tissue homeostasis in normal development

**Fig. 5:**
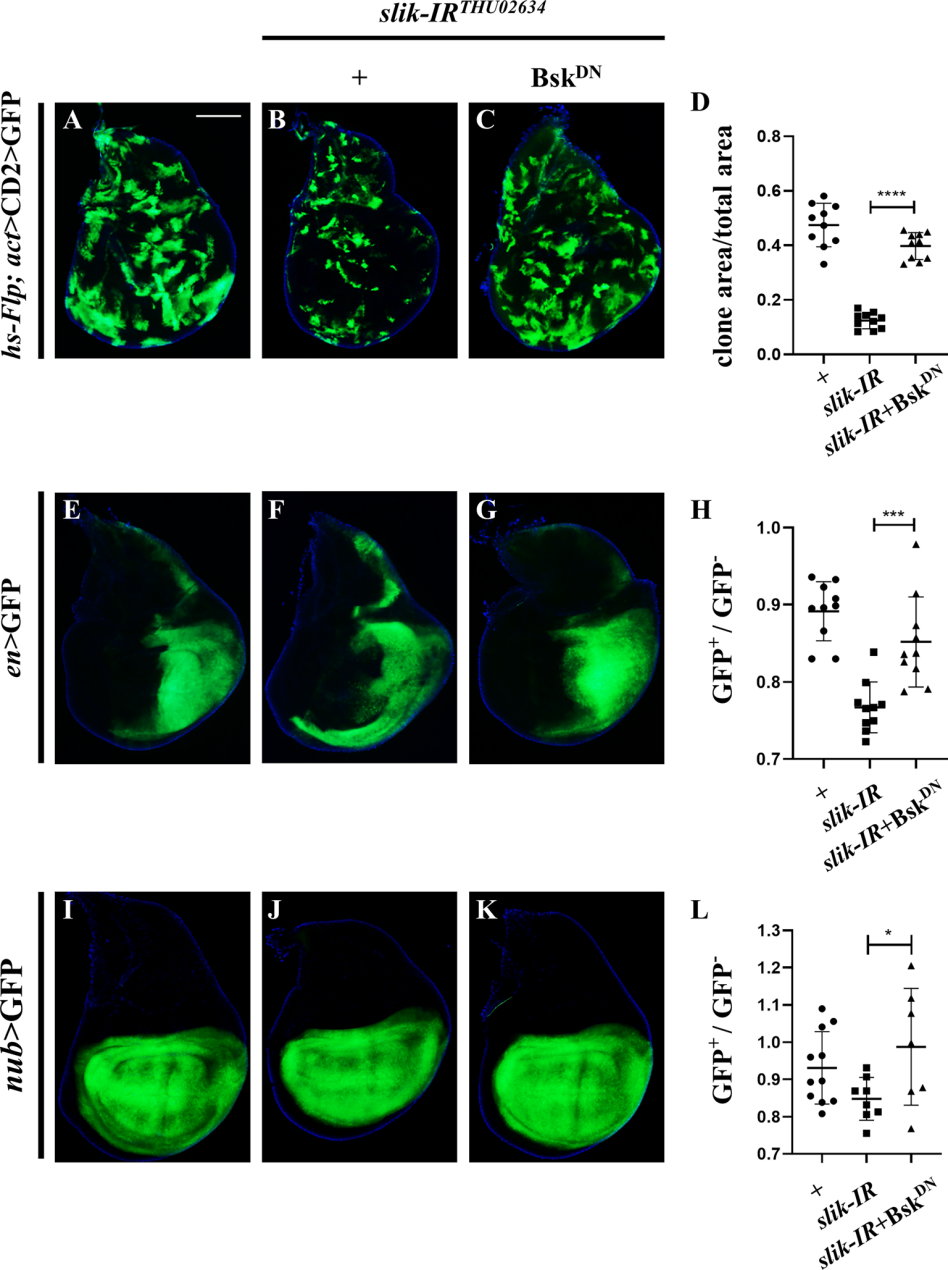
*slik* is physiologically required for proper tissue homeostasis in development. Merged fluorescence micrographs of *Drosophila* third instar larval wing discs are shown (**A**–**C**, **E**–**G**, **I–K**). From the Flp-out clones (marked by GFP), compared with the control (**A**), *slik* knockdown clones (GFP^+^) displayed decreased clonal size (**B**), which was rescued by expressing Bsk^DN^ (**C**). Statistical analysis of clone size/total size ratio in **A**–**C** are shown (**D**, n = 10 for each group). Besides, compared with the *en*-Gal4 (**E**) and *nub*-Gal4 controls (**I**), *slik* depletion-evoked decreased GFP area in the posterior compartment **F** or the whole wing pouch **J** was restored by Bsk^DN^ (**G**, **K**). Statistical analysis of GFP^+^ area/GFP^−^ area ratio in **E**–**G** (**H**, n = 10 for each group) and **I-K **(**L**, n = 11; n = 8; n = 7) are shown. One-way ANOVA with Bonferroni multiple-comparison test was used to compute P-values, ****P < 0.0001, ***p < 0.001, *P < 0.05, n.s indicates not significant. See the supplementary material for detailed genotypes. Scale bar: 100 μm

Strict regulation of cell death, which contributes to the adjustment of cell number and elimination of unwanted cells, is vital for tissue homeostasis and organism fitness [49]. To characterize the physiological function of *slik* in tissue homeostasis, we generated *RNAi*-mediated knockdown clones in the wing discs by the FLP/FRT-mediated recombination. Compared with the control clones, *slik* depletion resulted in decreased clone (GFP^+^) sizes and numbers, which were appreciably restored by expressing Bsk^DN^ (Fig. 5A–D, Additional file 1: Fig. S3A). Furthermore, *slik* knockdown either in the posterior compartment by *en*-Gal4 or in the wing pouch by *nub*-Gal4 resulted in diminished sizes of corresponding areas, which were largely recovered by blocking Bsk activity (Fig. 5E–L). Moreover, we also generated *slik* mutant clones by FLP/FRT-mediated genetic mosaic technique [50], and observed that *slik* mutant clones (GFP^−^) were significantly smaller than their simultaneously generated wild-type twin clones (2×GFP), while the control clones were almost the same size as their counterparts (Additional file 1: Fig. S3B–D). Together, these data suggest that *slik* is required to maintain JNK-dependent tissue homeostasis in normal development.

### Human STK10 rescues ***slik*** depletion-induced developmental defects in ***Drosophila***

**Fig. 6:**
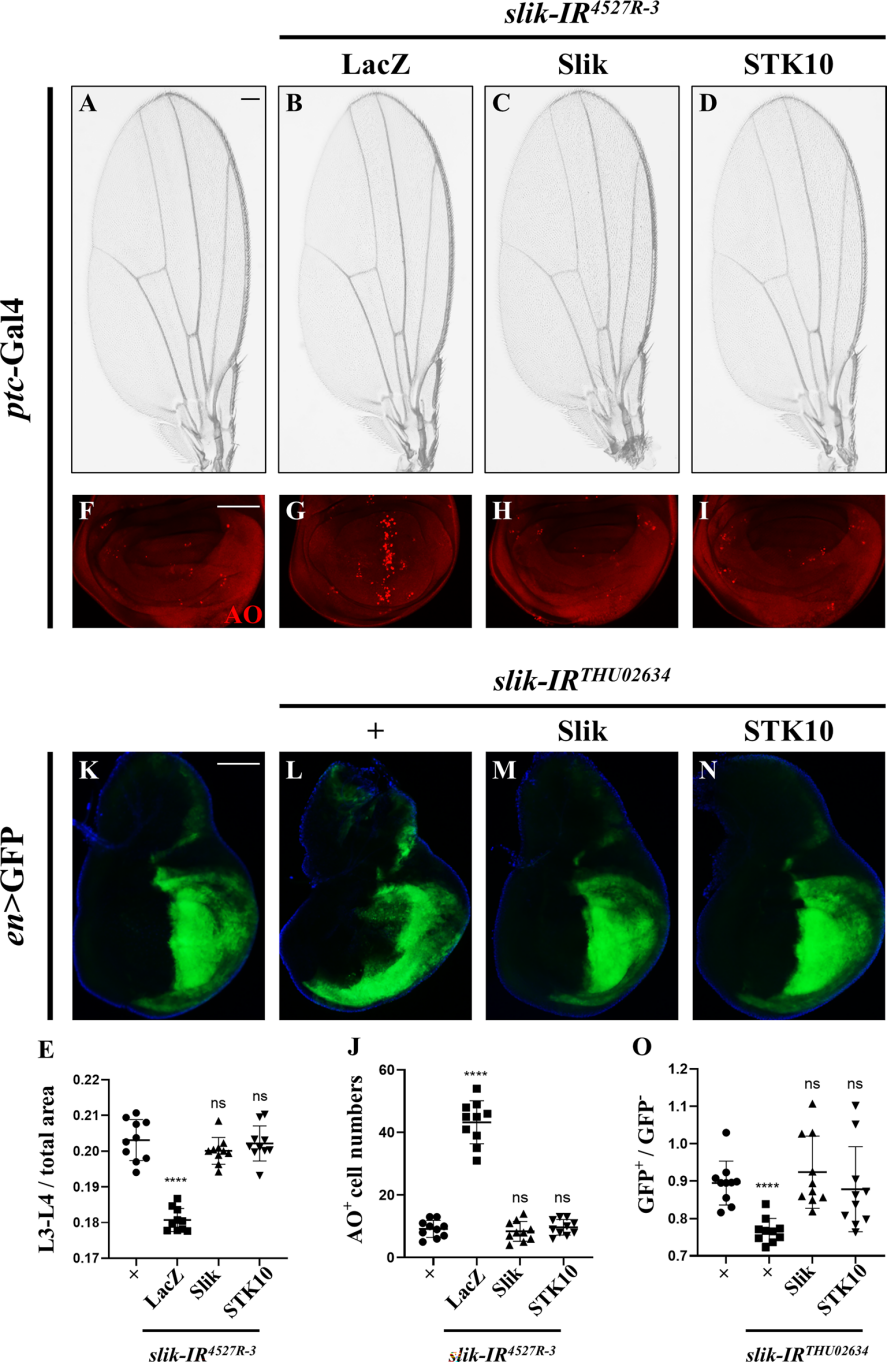
Human STK10 reliefs *slik* depletion-caused cell death. Light micrographs of adult wings (**A**–**D**) and fluorescence micrographs of third instar larval wing discs **F**–**I**, **K**–**N** are shown. Compared with the controls (**A**, **F**), loss of *slik* driven by *ptc*-Gal4 resulted in reduced L3-L4 area in adult wings **B** and intense AO staining in wing discs (**G**), which were effectively rescued by expressing Slik (**C**, **H**) or STK10 (**D**, **I**). Statistical analysis of the adult wing L3-L4 size/total size ratio (**E**, n = 10 for each genotype) and cell death number (**J**, n = 10 for each genotype) in wing discs are shown. Comparable with the *en*-Gal4 control (**K**), *slik* knockdown-induced GFP area reduction in the posterior compartment **L** was restored by expressing Slik **M** or STK10 **N**. Statistical analysis of GFP^+^ area/GFP^−^ area ratio in **K**-**N** is shown (**O**, n = 10 for each genotype). One-way ANOVA with Bonferroni multiple-comparison test was used to compute P-values, ****P < 0.0001, n.s indicates not significant. See the supplementary material for detailed genotypes. Scale bar: 100 μm

Slik encodes a member of the Sterile-20 kinase family, sharing 46% similarity and 32% identity with its human ortholog STK10. To check whether STK10 has retained the developmental functions of Slik in *Drosophila*, we produced *UAS*-STK10 transgenic flies and checked whether expression of STK10 could rescue *slik* loss-caused developmental defects. We noted that ectopic expression of Slik or STK10, verified by RT-qPCR (Additional file 1: Fig. S4), could largely rescue *ptc* > *slik-IR*-induced L3-L4 area reduction in adult wings (Fig. 6A–E) and robust cell death along A/P compartment boundary in larval wing discs (Fig. 6F–J). In addition, area reduction in the posterior compartment of *en > slik-IR* wing discs was efficiently rescued by expressing Slik or STK10 (Fig. 6K–O). Furthermore, MARCM analyses showed that the augmented apoptosis and diminished size of *slik* mutant clones (marked by GFP) were effectively mitigated upon the expression of Bsk^DN^, Slik, or STK10 (Additional file 1: Fig. S5). Thus, these data demonstrate that the developmental functions of Slik are evolutionarily conserved by STK10.

### Slik function in the JNK pathway is retained by STK10

**Fig. 7:**
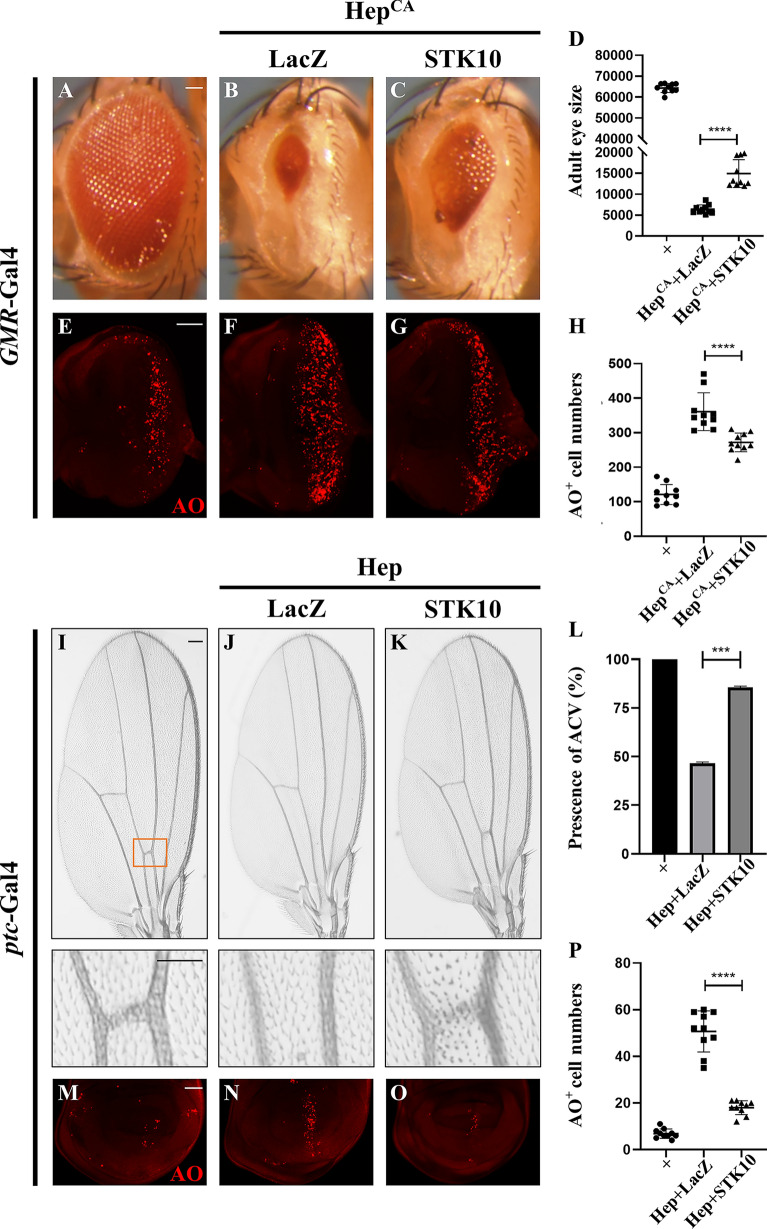
Human STK10 inhibits JNK signaling in *Drosophila*. Light micrographs of *Drosophila* adult eyes (**A**–**C**) and wings (**I**–**K**), and fluorescence micrographs of third instar larval eye (**E**–**G**) and wing discs (**M**–**O**) are shown. Compared with the controls (**A**, **E**), *GMR* > Hep^CA^-evoked small eye phenotype (**B**) and cell death in eye discs (**F**) were inhibited by expressing STK10 (**C**, **G**). Statistical analysis of the adult eye sizes (**D**, n = 10 for each genotype) and AO-positive cell numbers in eye discs (**H**, n = 10 for each genotype) are shown. Consistently, compared with the *ptc*-Gal4 controls (**I**, **M**), ectopic Hep induced a loss-of-ACV phenotype in adult wings (**J**) and robust cell death in wing discs (**N**), both of which were suppressed by expressing STK10 (**K**, **O**). The lower panels show high magnification view of the boxed areas in upper panels (**I**–**K**). The presence of ACV in adult wings (**L**, n = 15 for each genotype) and AO positive cell numbers in wing discs (**P**, n = 10 for each genotype) were quantified. One-way ANOVA with Bonferroni multiple-comparison test was used to compute P-values, ****p < 0.0001, ***p < 0.001. See the supplementary material for detailed genotypes. Scale bar: 50 μm in **A**–**C**, **E**–**G**, **I**–**K** (lower panels) and **M-O**, 100 μm in **I**–**K** (upper panels)

As the JNK pathway is evolutionarily conserved from fly to human, and STK10 is able to rescue *slik* loss-induced developmental defects, it is plausible that STK10 has retained the regulatory role of Slik in the JNK pathway. Consistently, *GMR* > Egr-triggered cell death in the larval eye discs and the small eye phenotype in the adults were effectively restored by expressing STK10, while *hep* depletion was included as a positive control (Additional file 1: Fig. S6). Since Egr triggers both JNK-dependent and JNK-independent cell death in development [12, 35, 51], to determine whether STK10 modulates JNK-mediated cell death, we activated JNK signaling by overexpressing the JNK kinase Hep. We found that expression of STK10 markedly inhibited *GMR* > Hep^CA^-caused small eye in adults (Fig. 7A–D) and increased AO staining in larval eye discs (Fig. 7E–H), and *ptc* > Hep-induced loss-of-ACV phenotype in adult wings (Fig. 7I–L) and cell death in larval wing discs (Fig. 7M–P). Hence, we conclude that STK10 could functionally substitute for Slik to regulate JNK-dependent cell death in *Drosophila*.

### Slik and STK10 modulates physiological JNK-mediated cell death

**Fig. 8:**
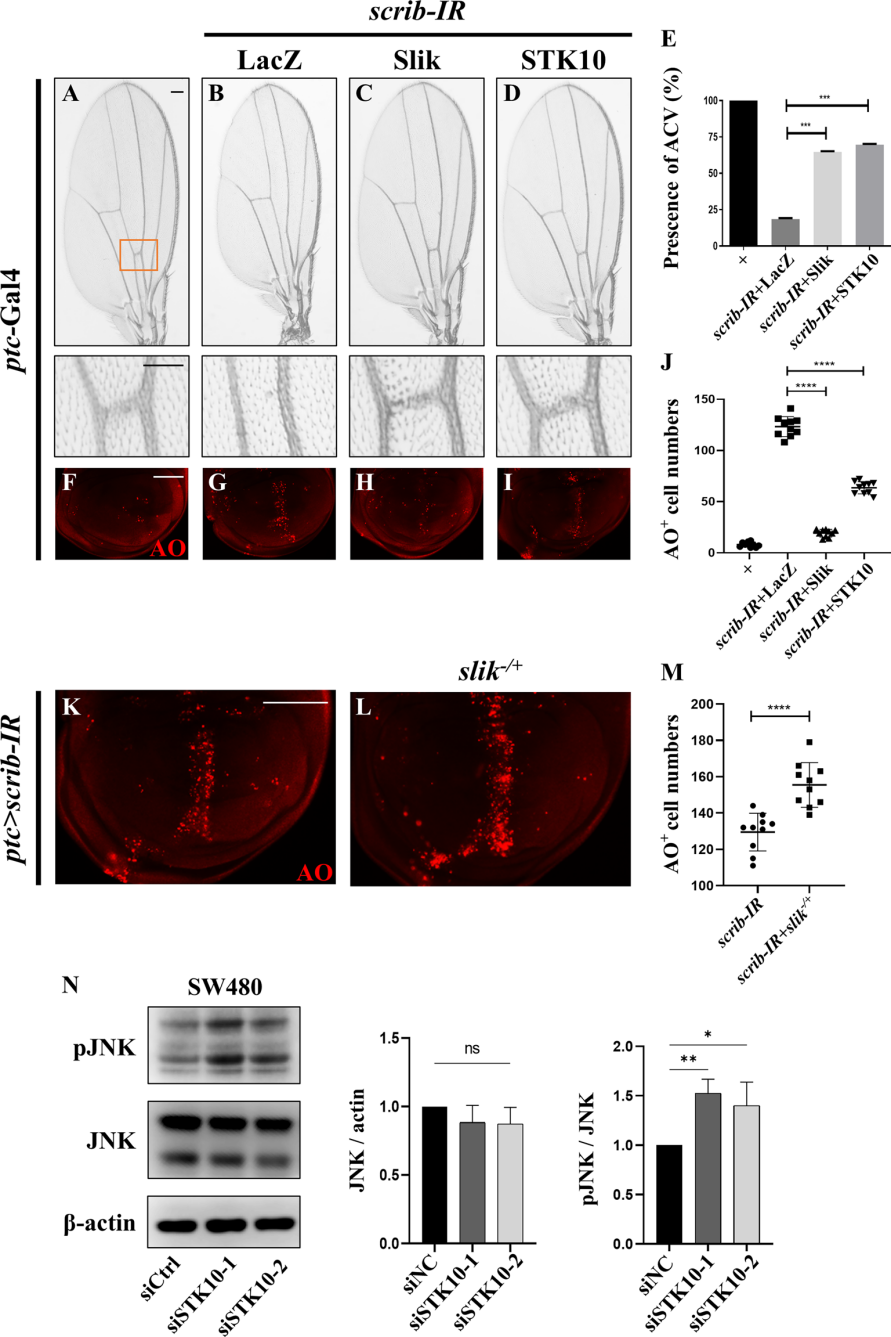
Slik/STK10 prevents physiological JNK-mediated cell death. Light micrographs of *Drosophila* adult wings (**A**–**D**) and fluorescence micrographs of third instar larval wing discs (**F**–**I**, **K**–**L**) are shown. RNAi-mediated down-regulation of *scrib* along the A/P compartment boundary by *ptc*-Gal4 triggered the loss-of-ACV phenotype in adult wings (**B**), which was resulted from cell death in larval wing discs (**G**). Both phenotypes were inhibited by expressing Slik or STK10 (**C**–**D**, **H**–**I**). The bottom panels show high magnification views of the boxed areas in upper panels (**A**–**D**). Importantly, *ptc > scrib-IR*-induced cell death (**K**) was enhanced in heterozygous *slik* mutants (**L**). Statistical analysis of the presence of ACV in adult wings (**E**, n = 15 for each genotype) and cell death number in wing discs (**J** and **M**, n = 10 for each genotype) were shown. (**N**) Immunoblot analysis of p-JNK and total JNK protein level in SW480 cells treated by nonspecific siRNA (siCtrl) or two independent siRNA targeting *STK10* (referred to as siSTK10-1 and siSTK10-2). p-JNK protein level was increased upon *STK10* knockdown, while total JNK protein level remained unchanged. One-way ANOVA with Bonferroni multiple-comparison test and unpaired two tailed t-test were used to compute P-values, ****p < 0.0001, ***p < 0.001, **p < 0.01, *P < 0.05. See the supplementary material for detailed genotypes. Scale bar: 100 μm in **A**–**D** (upper panels), **F**–**I** and **K**–**L**, 50 μm in **A**–**D** (lower panels)

The above results suggest that Slik negatively regulates ectopic JNK-mediated cell death, triggered by overexpression of Egr or Hep, yet it remains unknown whether Slik modulates the physiological JNK signaling. It has been reported that disruption of cell polarity promotes JNK-mediated cell death in development [52, 53]. Consistently, knockdown of the cell polarity gene *scrib* along the A/P compartment boundary by *ptc*-Gal4 induced severe cell death in wing discs (Fig. 8F, G) and ACV loss in adult wings (Fig. 8A, B). These phenotypes, caused by the activation of endogenous JNK signaling [5], were effectively inhibited by expressing Slik or STK10 (Fig. 8C–E, H–J), while expression of Bsk^DN^ and Puc served as the positive controls (Additional file 1: Fig. S7). Importantly, *ptc* > *scrib-IR*-increased cell death was potently aggravated in heterozygous *slik* mutants (Fig. 8K–M). Together, these results indicated that Slik modulates physiological JNK signaling-induced cell death in development.

Finally, to examine whether STK10 regulates JNK activity in human cells, we knockdown *STK10* in SW480 human colon cancer cells by two independent siRNA, whose efficacies were verified by RT-qPCR (Additional file 1: Fig. S8). We found that p-JNK level was up-regulated whereas total JNK protein level remained unaffected upon *STK10* knockdown (Fig. 8N). Intriguingly, *STK10* depletion-augmented p-JNK level was effectively mitigated by the expression of Slik, but not its kinase-deficient variant (Additional file 1: Fig. S9). These observations provide further evidence supporting the notion that Slik/STK10 play a conserved role in regulating the JNK pathway across both *Drosophila* and human cells, and the kinase activity of Slik/STK10 likely being crucial for this function.

## Discussion

Control of tissue growth requires coordinate actions of cell proliferation, growth and death [54, 55]. Cell death modulates a great deal of physiological processes, including regulation of cell number, sculpting of tissue structures, and elimination of abnormal or aged cells, which plays a fundamental role in tissue homeostasis and organ development [56, 57]. JNK pathway has been shown to play a critical role in tissue homeostasis by maintaining the proper balance of cell death and proliferation [58, 59]. In this study, we took advantage of the fly genetics and identified Slik, the *Drosophila* ortholog of mammalian STK10, as an essential regulator of JNK-mediated apoptosis. Consistent with our data, *slik* exhibits moderately high expression in 3rd instar larval imaginal discs [60]. In particular, loss of *slik* activates JNK signaling and promotes JNK-dependent apoptotic cell death, while overexpression of Slik or its human ortholog STK10 significantly suppresses ectopic or physiologic JNK-induced cell death in development. Furthermore, our genetic epistasis analysis established that Slik acts upstream of or in parallel to Hep, to regulate the JNK signaling in cell death (Additional file 1: Fig. S10). Notably, knockdown of *STK10* in human cells also results in JNK activation, suggest the inhibitory role of Slik/STK10 on JNK signaling has been evolutionarily conserved from fly to human.

Previous studies have revealed Moesin and Merlin as the main substrates of Slik. In addition, STRIPAK is shown to act as an upstream regulator that dephosphorylates Slik, which facilitates its association with the cortex [30]. In this work, we characterized Slik as a negative regulator of JNK pathway, yet the direct target of Slik in JNK signaling remains to be identified. It is worth noting that a previous study has documented the involvement of Moesin in the regulation of JNK-dependent cell death via the Rho1-Slpr-dTAK1 axis [61]. Notably, the inhibition of cell death induced by Moesin was observed upon the knockdown of dTAK1 [61], but no such effect was observed in the case of *slik* loss-triggered cell death (Fig. 4F, K). Therefore, it can be inferred that Slik may regulate JNK-mediated cell death through a distinct mechanism that is independent of Moesin.

As Slik encodes a kinase, Slik-mediated phosphorylation may suppress JNK activity via two possible mechanisms, activating a negative regulator or inactivating a positive regulator. It is well known that phosphorylation can either activate or inactivate a substrate protein. Intriguingly, Slik-mediated phosphorylation promotes the function of Moesin, but inhibits that of Merlin, which results in a coordinate regulation of proliferation and epithelial integrity [27, 31]. If Slik acts upstream of Hep, a possible mechanism is that Slik phosphorylates and activates an inhibitor of Hep, thus inhibits Hep-mediated JNK activation. The protein phosphatase PP2A has been shown to inactivate JNK signaling by dephosphorylating MKK7, the ortholog of Hep, in hepatocellular carcinoma (HCC) cells [61]. Interestingly, Cka, a regulatory subunit of PP2A, acts upstream of Hep to inhibit JNK signaling during *Drosophila* spermatogenesis [62]. Furthermore, a Cka-Slik physical interaction has been reported, which contributes to PP2A-mediated Slik dephosphorylation [30]. Thus, it is likely that Slik, in a feed-back manner, phosphorylates and activates Cka, which dephosphorylates Hep and inhibits JNK signaling. If Slik acts in parallel to Hep, a possible target is Puc, a serine/threonine protein phosphatase for Bsk. Kinase-mediated phosphorylation has been reported to promote phosphatases activities. For instance, the extracellular signal-regulated kinase 2 (ERK2) activates MAP kinase phosphatase-3 (MKP-3) [63], and protein kinase A (PKA) activates protein phosphatase 2 A (PP2A) by phosphorylation [64]. Of particular interest, mTORC2-induced phosphorylation on DUSP10, the human ortholog of Puc, blocks its proteasome-mediated degradation [65]. Hence, it is plausible that Slik/STK10 may inhibit JNK signaling via phosphorylation and activation of Puc/DUSP10. Apart from this, while Slik regulates Moesin and Merlin through its kinase function, a kinase independent genetic interaction of Slik with Raf has also been described [32, 66].

## Conclusions

Loss of *slik* triggers JNK-mediated apoptosis and impaired tissue homeostasis. Slik is necessary and sufficient for preventing physiological JNK-mediated apoptosis. Human STK10 rescues *slik* loss-induced cell death and impaired tissue homeostasis. Knockdown of STK10 in human cancer cells also leads to JNK activation, which is cancelled by expressing Slik. Thus, the inhibitory role of Slik/STK10 on JNK signaling has been evolutionarily conserved from fly to human.

## Materials and methods

### ***Drosophila*** strains

Flies were reared on a standard cornmeal and brown sugar medium at 25 °C unless otherwise indicated. The following strains were used in this work: *ptc*-Gal4, *en*-Gal4, *nub*-Gal4, *GMR*-Gal4, *UAS*-GFP [67], *TRE-RFP* [68], *UAS*-Egr, *UAS-*Hep [53], *UAS*-Puc, *UAS*-Bsk^DN^ [69], *UAS-scrib-IR*, *UAS*-P35 [34], *yw hs*-*Flp*; *act* > CD2 > Gal4 *UAS*-GFP [70], *UAS*-*dTRAF2*-*IR, UAS-dTAK1-IR* [14] and *UAS*-*bsk*-*IR* [71] were previously described. *UAS*-*slik*-*IR* (55,626), *UAS-hep-IR* (28,710), *UAS-*Hep^CA^ (6406), *Df(2R)BSC603* (25,436), *Df(2R)ED4065* (9069), *Df(2R)ED4071* (24,117), *UAS*-LacZ (3956) and *hs*-Gal4 (1799) were obtained from the Bloomington stock center, *UAS*-*egr*-*IR* (45,253) was acquired from the Vienna Drosophila RNAi Center, *UAS*-*slik*-*IR* (4527R-3) was received from Japanese National Institute of Genetics (NIG), FRT42D *slik*^*KG04837*^ (114,386) was obtained from Kyoto Stock Center, *UAS*-*slik*-*IR* (02634) was got from Tsing Hua Fly Center. *yw hs*-*Flp*; FRT42D *ubi*-GFP was provided by professor Haiyun Song, FRT42D was provided by professor Xianjue Ma, *hs*-*Flp*; FRT42D *tub*-Gal80; *tub*-Gal4 *UAS*-GFP was provided by professor Chenhui Wang. The human STK10 and *Drosophila* Slik expression plasmid, generated by PCR and subcloned into PUAST vector, was further used to produce the transgenic flies.

### Acridine Orange (AO) staining

Eye and wing discs were dissected from third-instar larvae in 0.1% PBST (phosphate-buffered saline + 0.1% Tween-20) and incubated in 1×10^-5^ M AO for 5 min at room temperature prior to imaging.

### Immunostaining

Antibody staining was performed by standard procedures for imaginal discs. Dissected discs were fixed in 4% formaldehyde for 20 min. After several washes with 0.3% (v/v) PBST, discs were stained overnight with primary antibodies at 4 ℃, then washed with PBST and incubated with the secondary antibody for 2 h at room temperature. The following primary antibodies were used for immunostaining: rabbit anti-Cleaved Dcp-1 (Cell Signaling Technology, 9578, 1:100), rabbit anti-phospho-JNK (Calbiochem, 559,309, 1:200), secondary antibody is goat anti-rabbit CY3 (Life technologies, A10520, 1:1000).

### Heat shock induction of clones

Fluorescently labelled clones were produced in larval imaginal discs using the *yw hs*-*Flp*; *act* > CD2 > Gal4 *UAS*-GFP strain. Clones were induced by heat shock at 37 °C for 30 min, late third-instar larvae were dissected after recovering for 4 days (Fig. 5A–C).

Fluorescently labelled *slik* mutant MARCM clones were generated by *hs*-*Flp*; FRT42D *tub*-Gal80; *tub*-Gal4 *UAS*-GFP strain and clones were created by heat shock at 37 °C for 30 min, late third-instar larvae were dissected after recovering for 4 days (Additional file 1: Fig. S5). Twinspot clones in larval imaginal discs using *yw hs*-*Flp*; FRT42D *ubi*-GFP were induced by heat shock at 37 °C for 30 min, recovering for 3 days (Additional file 1: Fig. S3).

### RT-qPCR

For RNAi-knockdown efficiency experiments, *hs*-Gal4 driver was used. Animals were raised at 25 °C, heat-shocked at 37 °C for 30 min, and recovered at 29 °C for 2 h before dissection.

Eastep Super (Shanghai Promega) was used to isolate total RNA from third instar larvae of indicated genotypes, and RT-qPCR was performed using SYBR Green PCR Premix Kit (TaKaRa). Primers used were as follows:

*rp49* FP: CCACCAGTCGGATCGATATGC.

*rp49* RP: CTCTTGAGAACGCAGGCGACC.

*slik* FP: TAGGACAGCAGCAATGAGCTGG.

*slik* RP: TTCACGTAGCTCCTGCTTACGG.

*STK10* FP: ATCCTGCGCCTGTCTACCTT.

*STK10* RP: GCCTTGTAAACCTTGCCGAA.

### Cell culture and transfection

Human cells were cultured at 37 °C in DMEM (Gibco) containing 10% FBS, 100 U/mL penicillin, and 100 µg/mL streptomycin in a humidified incubator with 5% CO2. Lipofectamine RNAiMAX (Invitrogen, 13,778,150) was used for small-interfering RNA (siRNA) transfection as instructed. For *STK10* knockdown, two different siRNAs were used. The sequences are as follows:

siRNA-STK10-1: 5′-AGGAGGAGCUGGAGGACUATT-3′ (sense);

siRNA-STK10-1: 5′-UAGUCCUCCAGCUCCUCCUTT-3′ (antisense);

siRNA-STK10-2: 5′-CGACAGCAGCGGAAGGAAATT-3′ (sense);

siRNA-STK10-2: 5′-UUUCCUUCCGCUGCUGUCGTT-3′ (antisense);

siRNA-Control: 5′-UUCUCCGAACGUGUCACGUTT-3′ (sense);

siRNA-Control: 5′-ACGUGACACGUUCGGAGAATT-3′ (antisense).

### Immunoblotting

Cells were harvested and washed in ice-cold PBS, then lysed with RIPA lysis buffer (WELLBIO, WB0101, China) supplemented with protease inhibitor cocktails (Yeasen, 20124ES03, China) on ice for 30 min. Cell lysates were then centrifuged at 15,000 rpm for 10 min at 4 °C. Proteins were separated by SDS-PAGE following standard procedures. The primary antibodies were used as follows: Rabbit anti-β actin (CST, 8457, 1:2000), Rabbit anti-SAPK/JNK (CST, 9252, 1:2000) and Rabbit anti-phospho SAPK/JNK (CST, 9251, 1:2000).

### Image of fly wings and eyes

Three-day-old flies were collected and frozen at − 80 °C. When taking pictures, flies were unfrozen at room temperature and placed on 1% agarose plate. Light images of eyes were taken by OLYMPUS stereo microscope SZX16 (Olympus Corporation, Shinjuku, Tokyo, Japan). Light images of wings, dissected and placed on slide with alcohol/glycerol (1:1) medium, were taken by OLYMPUS BX51 microscope.

### Statistical analysis

Statistical analysis was performed with GraphPad Prism 8.0.2 software. The data were analyzed by One-way ANOVA with Bonferroni’s multiple comparison test and two-tailed unpaired t-test to calculate statistical significance (*P < 0.05; **P < 0.01; ***P < 0.001, ****P < 0.0001; ns, no significant difference). Error bar indicates standard deviation. The experiments were repeated at least three times.

### Supplementary Information

**Additional file 1: Figure S1.** A genetic screen for modifiers of GMR>Egr-induced eye-ablation phenotype. **A** A schematic depiction of the three deficiencies Df(2R)BSC603, Df(2R)ED4065, and Df(2R)ED4071. **B**–**G** Light micrographs of adult eyes. GMR>Egr-induced small eye phenotype (**B**) was obviously enhanced by deficiency Df(2R)BSC603 (**C**), Df(2R)ED4065 (**D**) or Df(2R)ED4071 (**E**), or by slikKG04837(**F**) or slik RNAi (**G**). Scale bar: 100 µm. **Figure S2.** Depletion of slik causes apoptotic cell death. Fluorescence micrographs of third instar larval wing discs are shown (**A**–**D**). Compared with the control (**A**), knockdown of slik driven by ptc-Gal4 led to massive cell death along the A/P compartment boundary (**B**), which was completely impeded by expression of P35 (**D**), but not that of lacZ-IR (**C**). Statistical analysis of AO positive cell number in wing discs (**E**, n=10 for each genotype) is shown. One-way ANOVA with Bonferroni multiple comparison test was used to compute P-values, ****P < 0.0001; ns, no significant difference. Scale bar: 40 µm. **Figure S3.** Slik is required for maintaining tissue homeostasis in development. **A** Statistical analysis of clone numbers in Fig. 5A-C are shown (n=10 for each group). **B**–**C** Fluorescence micrographs of third instar larval wing discs with twin clones (marked with 2ÍGFP or the absence of GFP) are shown. Compared with the control (**B**–**B’**), slik mutant clone (black) was obviously smaller than its wild-type twin clone (2ÍGFP) (**C**–**C’**). Statistical analysis of GFP-area/2ÍGFP area ratio (**D**, n=10 for each genotype) is shown. Unpaired two tailed t-test was used to compute P-values,****p < 0.0001. Scale bar: 25 µm. **Figure S4.** The efficacies of Slik and STK10 overexpression. mRNA level of slik (**A**) or STK10 (**B**) was measured by quantitative RT-PCR (n=2). Unpaired two tailed t-test was used to compute P-values, ****p < 0.0001, **p < 0.01. **Figure S5.** STK10 suppresses JNK-mediated apoptosis in slik mutant clones. **A**–**E** Fluorescence micrographs of third instar larval wing discs with MARCM clones (marked with GFP) are shown. Compared with the control (**A**-**A’’**), apoptosis was triggered in slik mutant clone (**B**-**B’’**), which was suppressed by the expression of Slik (**C**-**C’’**), STK10 (**D**-**D’’**) or BskDN (**E**-**E’’**). Scale bar: 25 µm. **Figure S6.** Human STK10 suppresses Egr-triggered cell death in eye development. Light micrographs of adult eyes (**A**–**D**) and fluorescence micrographs of third instar larval eye discs (**F**–**I**) are shown. Compared with the controls (**A**, **F**), GMR>Egr-induced small eye phenotype (**B**) and massive cell death in eye discs (**G**) were obviously suppressed by expressing STK10 (**C**, **H**), or hep-IR (**D**, **I**) serving as a positive control. Statistical analysis of adult eye size (**E**, n=10 for each genotype) and AO positive cell numbers in eye discs (**J**, n=10 for each genotype) are shown. One-way ANOVA with Bonferroni multiple-comparison test was used to compute P-values, ****p < 0.0001, ***p< 0.001, **p < 0.01. Scale bar: 100 μm. **Figure S7.** scrib depletion-induced cell death depends on JNK pathway. Light micrographs of adult wings (**A**–**C**) and fluorescence micrographs of third instar larval wing discs (**E**–**G**) are shown. ptc>scrib-IR-triggered loss-of-ACV phenotype in adult wings (**A**) and cell death in larval wing discs (**E**) were suppressed by expression of BskDN (B, F) or Puc (**C**, **G**). Statistical analysis of the presence of ACV in adult wings (**D**, n=15 for each genotype) and cell death number in wing discs (**H**, n≥10 for each genotype) are shown. One-way ANOVA with Bonferroni multiple-comparison test was used to compute P-values, ****p < 0.0001, **p < 0.01. Scale bar: 100 µm in **A**–**C** (upper panels), 50 µm in **A**–**C** (lower panels) and **E**–**G**.**Figure S8.** The knock-down efficacies of STK10 RNAi. Validation of two independent siRNA-STK10 used in current study. SW480 cells treated with siRNA for 72 hours were subjected to RT-qPCR (n=3). One-way ANOVA with Bonferroni multiple-comparison test was used to compute P-values, ****p < 0.0001. **Figure S9.** Kinase activity is necessary for Slik/STK10 to regulate JNK pathway. Immunoblot analysis of p-JNK and total JNK protein level in SW480 cells. Compared with the control (lane 1), STK10 knockdown increased p-JNK level (lane 2), which was effectively suppressed by expressing Slik (lane 3), but not a truncated Slik with kinase domain deletion (lane 4). One-way ANOVA with Bonferroni multiple-comparison test was used to compute P-values, ****p< 0.0001, ***p < 0.001. **Figure S10.** Schematic summary of Slik in regulating JNK pathway. Slik inhibits JNK-mediated cell death. Genetic epistasis analysis suggests that Slik acts upstream of or in parallel to Hep to impede JNK pathway.

## Data Availability

The datasets generated during the current study are available from the corresponding author on reasonable request.
