# Supplementary material for: Slik maintains tissue homeostasis by preventing JNK-mediated apoptosis
Source: Cell Div. 2023 Oct 4;18:16. doi: 10.1186/s13008-023-00097-4 (PMC10552427; doi:10.1186/s13008-023-00097-4)
Supplement: Supplementary file 1 — Additional file 1: Figure S1. A genetic screen for modifiers of GMR>Egr-induced eye-ablation phenotype. A A schematic depiction of the three deficiencies Df(2R)BSC603, Df(2R)ED4065, and Df(2R)ED4071. B–G Light micrographs of adult eyes. GMR>Egr-induced small eye phenotype (B) was obviously enhanced by deficiency Df(2R)BSC603 (C), Df(2R)ED4065 (D) or Df(2R)ED4071 (E), or by slikKG04837(F) or slik RNAi (G). Scale bar: 100 µm. Figure S2. Depletion of slik causes apoptotic cell death. Fluorescence micrographs of third instar larval wing discs are shown (A–D). Compared with the control (A), knockdown of slik driven by ptc-Gal4 led to massive cell death along the A/P compartment boundary (B), which was completely impeded by expression of P35 (D), but not that of lacZ-IR (C). Statistical analysis of AO positive cell number in wing discs (E, n=10 for each genotype) is shown. One-way ANOVA with Bonferroni multiple comparison test was used to compute P-values, ****P < 0.0001; ns, no significant difference. Scale bar: 40 µm. Figure S3. Slik is required for maintaining tissue homeostasis in development. A Statistical analysis of clone numbers in Fig. 5A-C are shown (n=10 for each group). B–C Fluorescence micrographs of third instar larval wing discs with twin clones (marked with 2ÍGFP or the absence of GFP) are shown. Compared with the control (B–B’), slik mutant clone (black) was obviously smaller than its wild-type twin clone (2ÍGFP) (C–C’). Statistical analysis of GFP-area/2ÍGFP area ratio (D, n=10 for each genotype) is shown. Unpaired two tailed t-test was used to compute P-values,****p < 0.0001. Scale bar: 25 µm. Figure S4. The efficacies of Slik and STK10 overexpression. mRNA level of slik (A) or STK10 (B) was measured by quantitative RT-PCR (n=2). Unpaired two tailed t-test was used to compute P-values, ****p < 0.0001, **p < 0.01. Figure S5. STK10 suppresses JNK-mediated apoptosis in slik mutant clones. A–E Fluorescence micrographs of third instar larval wing discs with [file 13008_2023_97_MOESM1_ESM.docx]

**Slik maintains tissue homeostasis by preventing JNK-mediated apoptosis**

Chenglin Li^1^, Xiaojie Zhu^1^, Xinyue Sun^1^, Xiaowei Guo^2^, Wenzhe Li^1^, Ping Chen^1^, Yulii V. Shidlovskii^3,4^, Qian Zhou^1,*^ and Lei Xue^1, 5,*^

^1^ The First Rehabilitation Hospital of Shanghai, Shanghai Key Laboratory of Signaling and Diseases Research, School of Life Science and Technology, Tongji University, Shanghai, China;

^2^ The Key Laboratory of Model Animals and Stem Cell Biology in Hunan Province, School of Medicine, Hunan Normal University, Changsha, Hunan, China;

^3^ Department of Gene Expression Regulation in Development, Institute of Gene Biology, Russian Academy of Sciences, Moscow, Russia;

^4^ Department of Biology and General Genetics, Sechenov University, 8, bldg. 2 Trubetskaya St., 119048 Moscow, Russia;

^5^ Zhuhai Precision Medical Center, Guangdong Provincial Key Laboratory of Tumor Interventional Diagnosis and Treatment, Zhuhai People's Hospital, Zhuhai Hospital Affiliated with Jinan University, Zhuhai, Guangdong, China.

- Correspondence: [lei.xue@tongji.edu.cn](mailto:lei.xue@tongji.edu.cn), [zhouqian80@163.com](mailto:zhouqian80@163.com)

**Supplementary Information**

Supplementary Figures

Detailed Genotypes

**
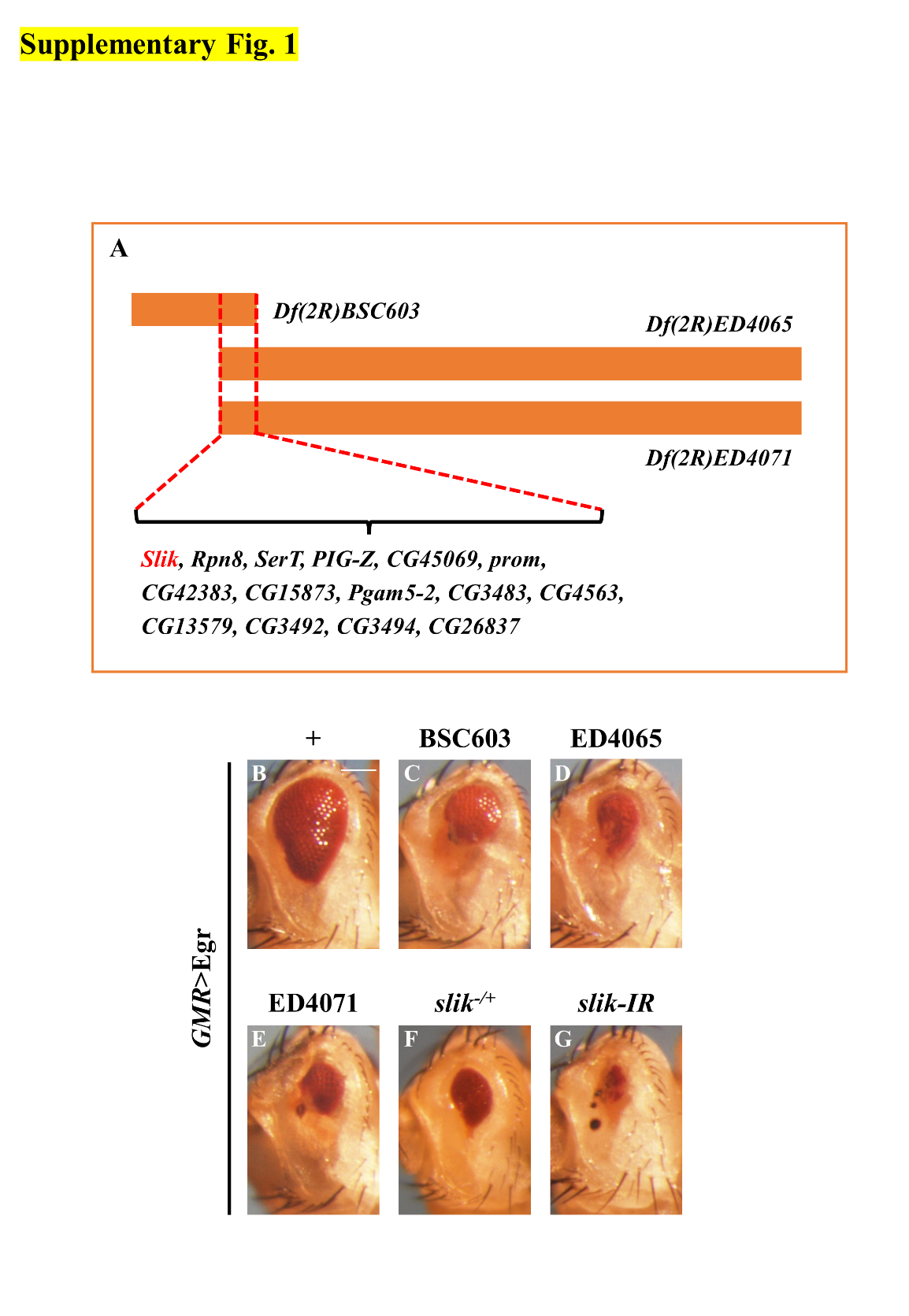
Li et al., Supplement Figure 1**

**Figure S1. A genetic screen for modifiers of *GMR*>Egr-induced eye-ablation phenotype**

(A) A schematic depiction of the three deficiencies *Df(2R)BSC603*, *Df(2R)ED4065*, and *Df(2R)ED4071*. (B-G) Light micrographs of adult eyes. *GMR*>Egr-induced small eye phenotype (B) was obviously enhanced by deficiency *Df(2R)BSC603* (C), *Df(2R)ED4065* (D) or *Df(2R)ED4071* (E), or by *slik^KG04837^* (F) or *slik* RNAi (G). Scale bar: 100 µm.

**
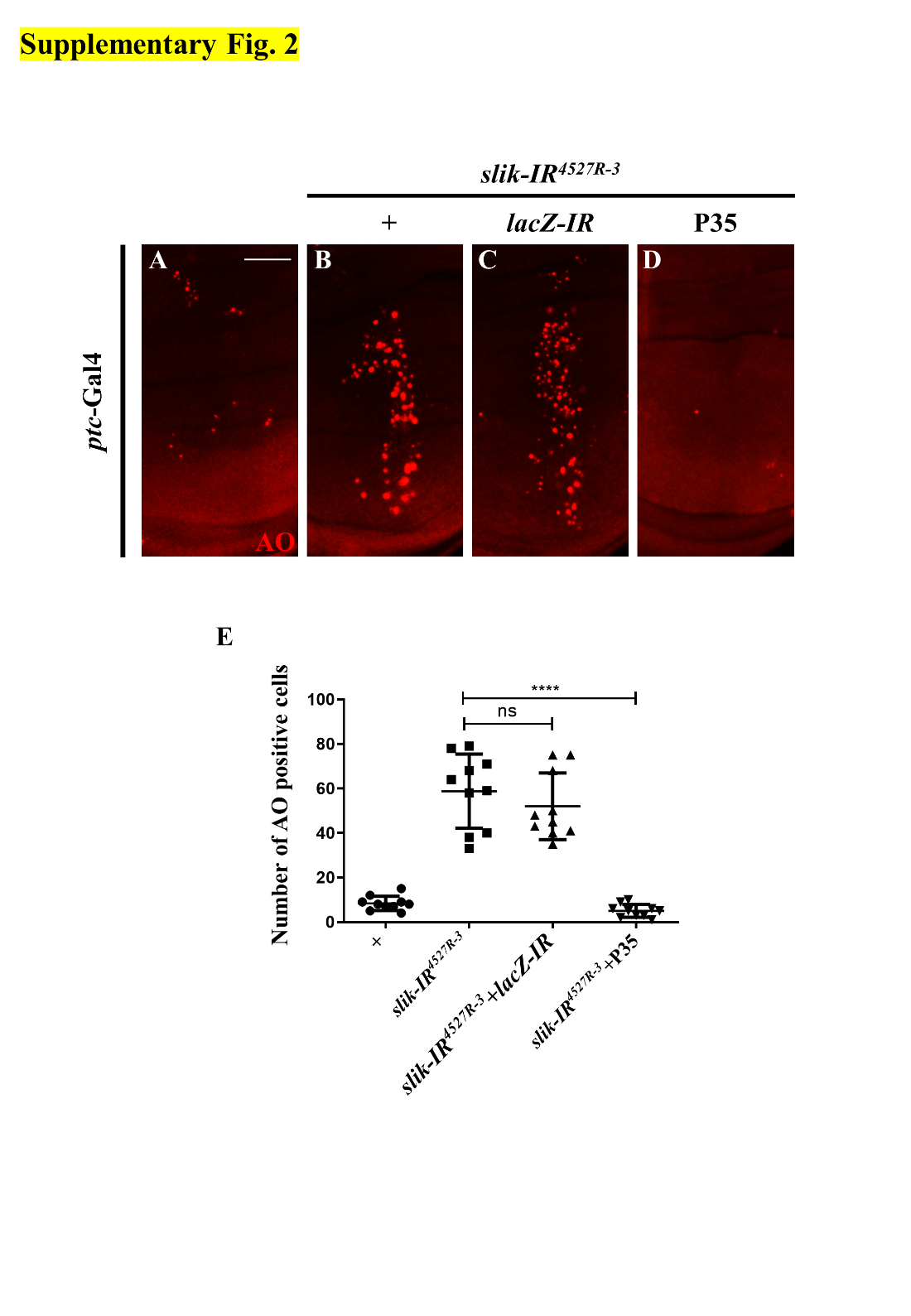
Li et al., Supplement Figure 2**

**Figure S2. Depletion of *slik* causes apoptotic cell death**

Fluorescence micrographs of third instar larval wing discs are shown (A-D). Compared with the control (A), knockdown of *slik* driven by *ptc*-Gal4 led to massive cell death along the A/P compartment boundary (B), which was completely impeded by expression of P35 (D), but not that of *lacZ-IR* (C). Statistical analysis of AO positive cell number in wing discs (E, n=10 for each genotype) is shown. One-way ANOVA with Bonferroni multiple comparison test was used to compute P-values, ****P < 0.0001; ns, no significant difference. Scale bar: 40 µm.

**
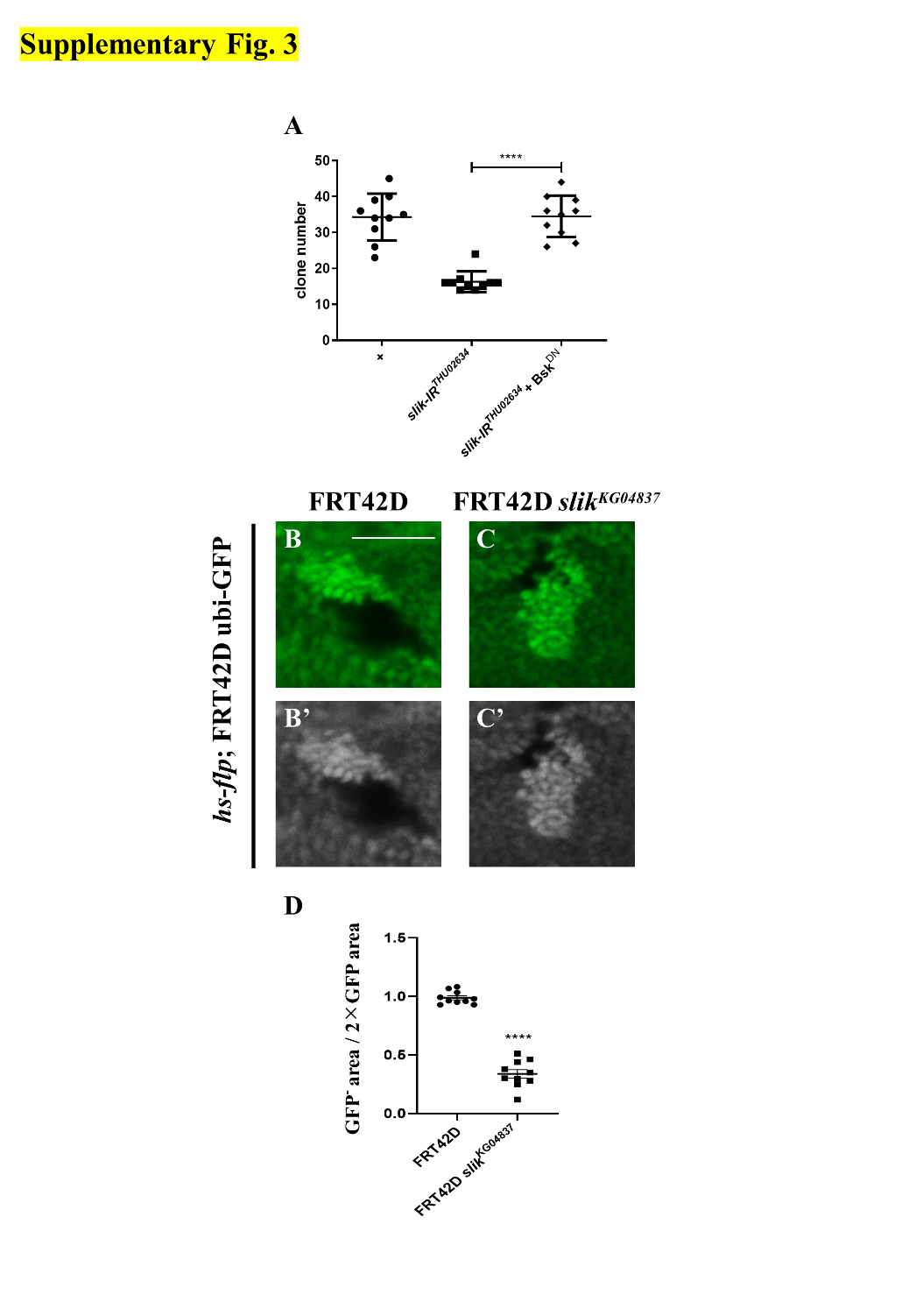
Li et al., Supplement Figure 3**

**Figure S3. *slik* is required for maintaining tissue homeostasis in development**

(A) Statistical analysis of clone numbers in Fig. 5A-C are shown (n=10 for each group).

(B-C) Fluorescence micrographs of third instar larval wing discs with twin clones (marked with 2🞨GFP or the absence of GFP) are shown. Compared with the control (B-B’), *slik* mutant clone (black) was obviously smaller than its wild-type twin clone (2🞨GFP) (C-C’). Statistical analysis of GFP^-^ area/2🞨GFP area ratio (D, n=10 for each genotype) is shown. Unpaired two tailed t-test was used to compute P-values, ****p < 0.0001. Scale bar: 25 µm.

**
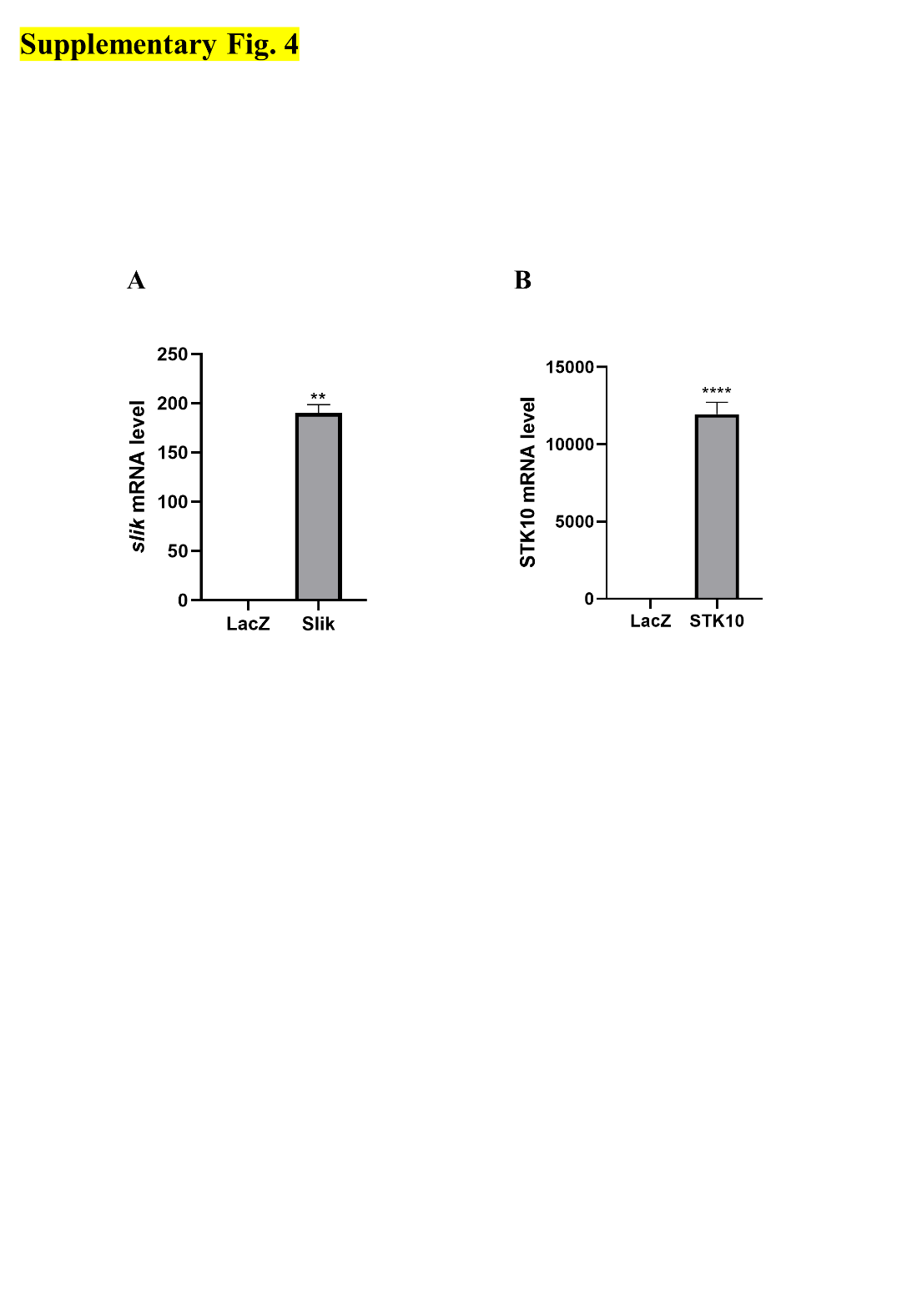
Li et al., Supplement Figure 4**

**Figure S4.** **The efficacies of Slik and STK10 overexpression**

mRNA level of *slik* (A) or *STK10* (B) was measured by quantitative RT-PCR (n=2). Unpaired two tailed t-test was used to compute P-values, ****p < 0.0001, **p < 0.01.

**
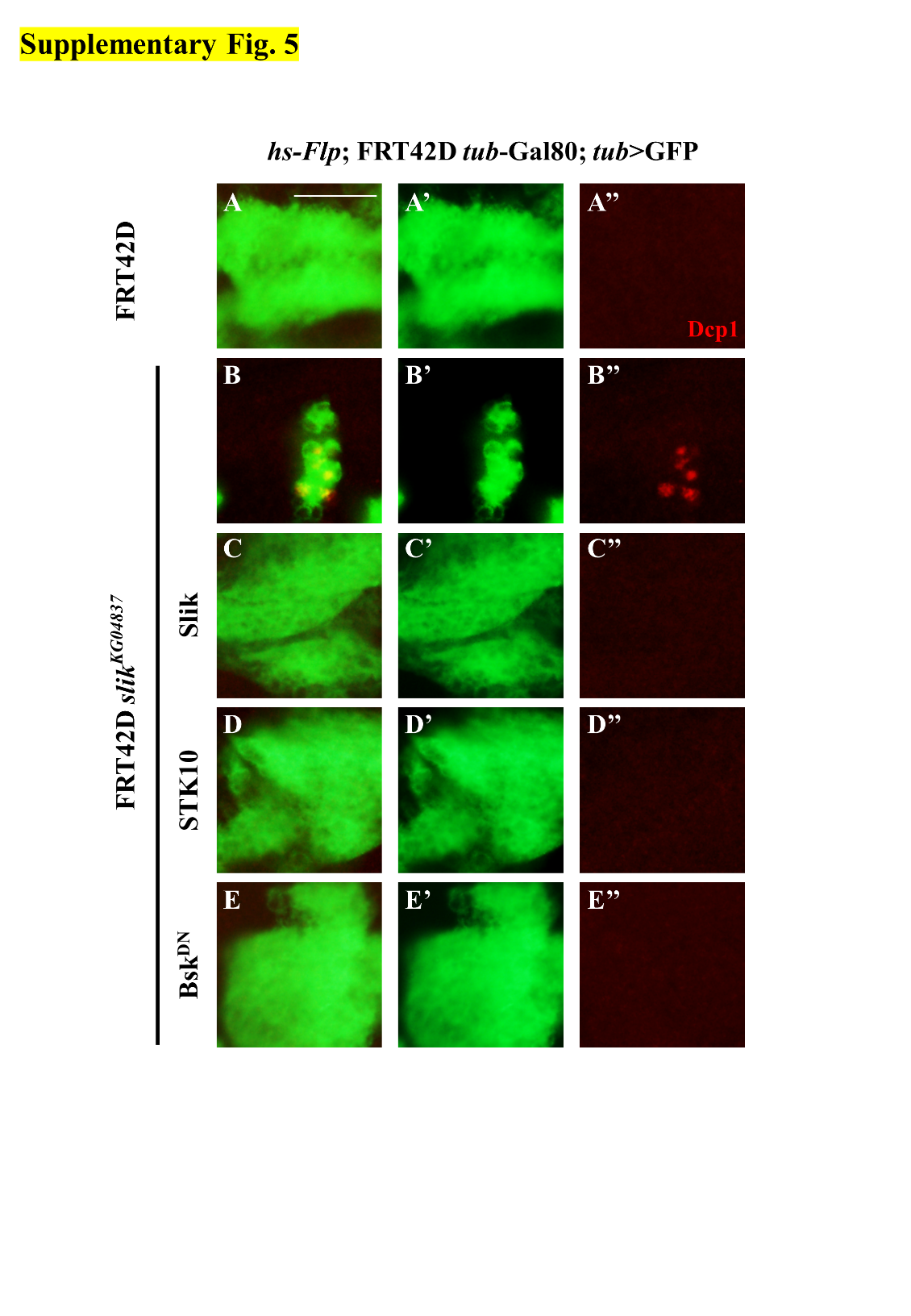
Li et al., Supplement Figure 5**

**Figure S5. STK10 suppresses JNK-mediated apoptosis in *slik* mutant clones**

(A-E) Fluorescence micrographs of third instar larval wing discs with MARCM clones (marked with GFP) are shown. Compared with the control (A-A’’), apoptosis was triggered in *slik* mutant clone (B-B’’), which was suppressed by the expression of Slik (C-C’’), STK10 (D-D’’) or Bsk^DN^ (E-E’’). Scale bar: 25 µm.

**
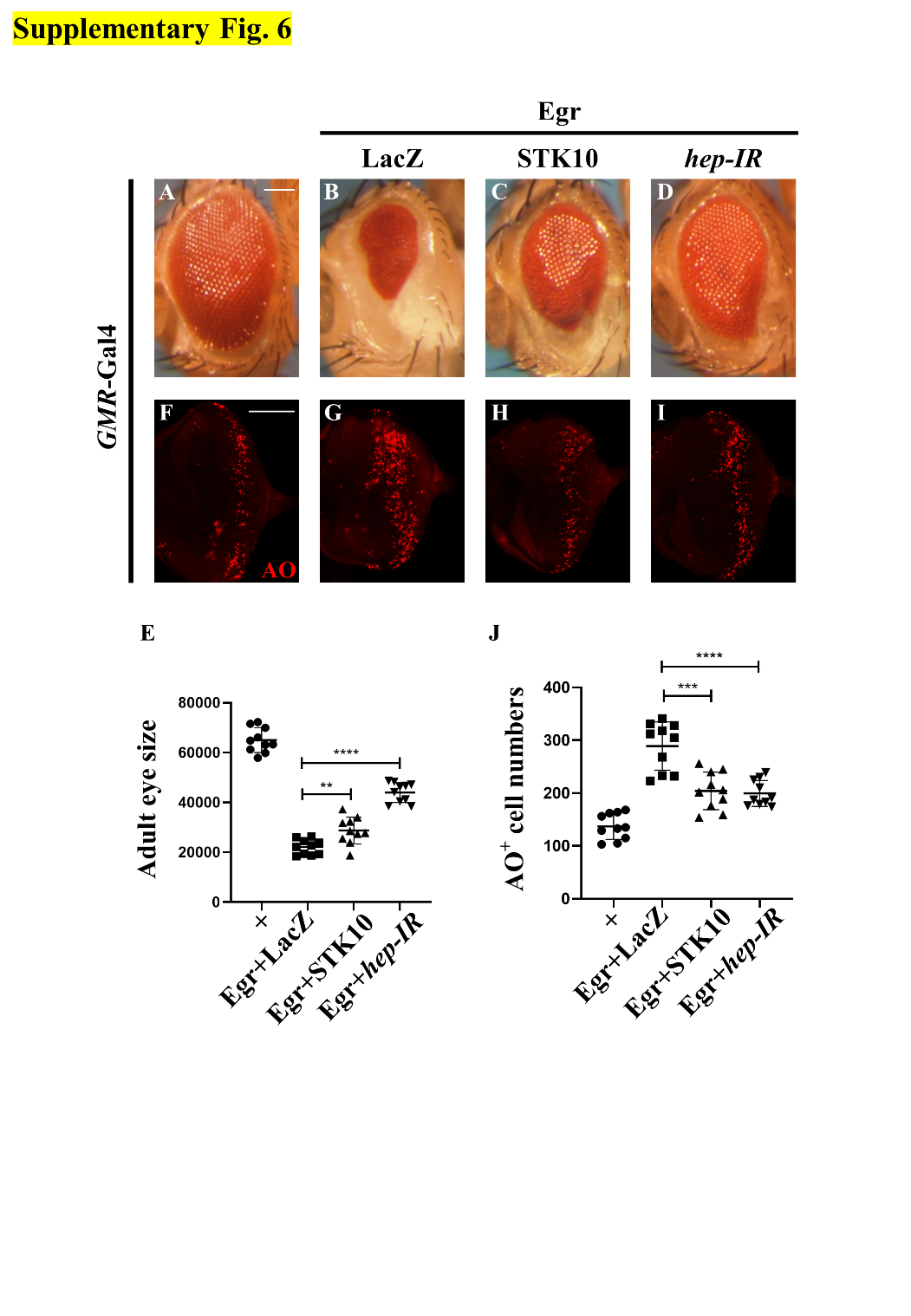
Li et al., Supplement Figure 6**

**Figure S6.** **Human** **STK10 suppresses Egr-triggered cell death in eye development**

Light micrographs of adult eyes (A-D) and fluorescence micrographs of third instar larval eye discs (F-I) are shown. Compared with the controls (A, F), *GMR*>Egr-induced small eye phenotype (B) and massive cell death in eye discs (G) were obviously suppressed by expressing STK10 (C, H), or *hep-IR* (D, I) serving as a positive control. Statistical analysis of adult eye size (E, n=10 for each genotype) and AO positive cell numbers in eye discs (J, n=10 for each genotype) are shown. One-way ANOVA with Bonferroni multiple-comparison test was used to compute P-values, ****p < 0.0001, ***p < 0.001, **p < 0.01. Scale bar: 100μm.

**
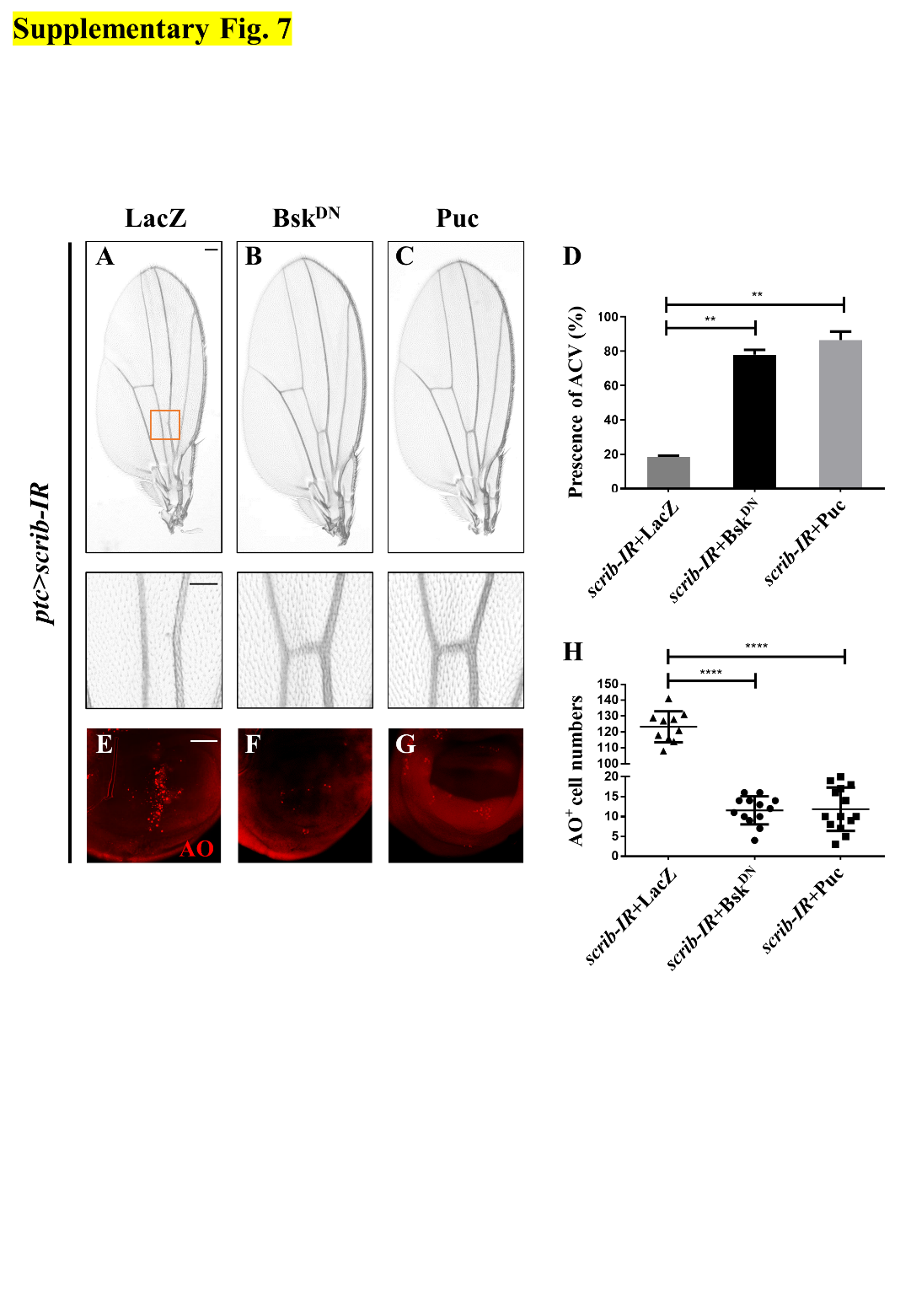
Li et al., Supplement Figure 7**

**Figure S7.** ***scrib* depletion-induced cell death depends on JNK pathway**

Light micrographs of adult wings (A-C) and fluorescence micrographs of third instar larval wing discs (E-G) are shown. *ptc*>*scrib-IR*-triggered loss-of-ACV phenotype in adult wings (A) and cell death in larval wing discs (E) were suppressed by expression of Bsk^DN^ (B, F) or Puc (C, G). Statistical analysis of the presence of ACV in adult wings (D, n=15 for each genotype) and cell death number in wing discs (H, n≥10 for each genotype) are shown. One-way ANOVA with Bonferroni multiple-comparison test was used to compute P-values, ****p < 0.0001, **p < 0.01. Scale bar: 100 µm in A-C (upper panels), 50 µm in A-C (lower panels) and E-G.

**Li et al., Supplement Figure 8**

**
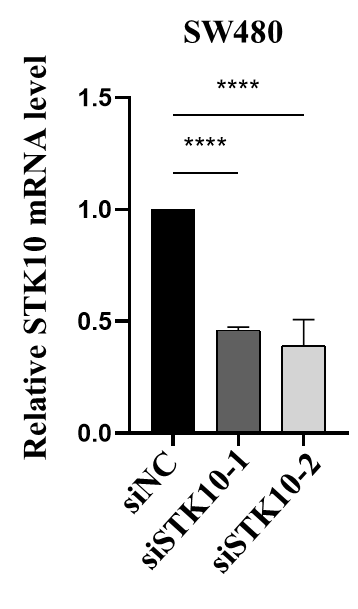
**

**Figure S8.** **The knock-down efficacies of *STK10 RNAi***

Validation of two independent siRNA-STK10 used in current study. SW480 cells treated with siRNA for 72 hours were subjected to RT-qPCR (n=3). One-way ANOVA with Bonferroni multiple-comparison test was used to compute P-values, ****p < 0.0001.

**Li et al., Supplement Figure 9**

**
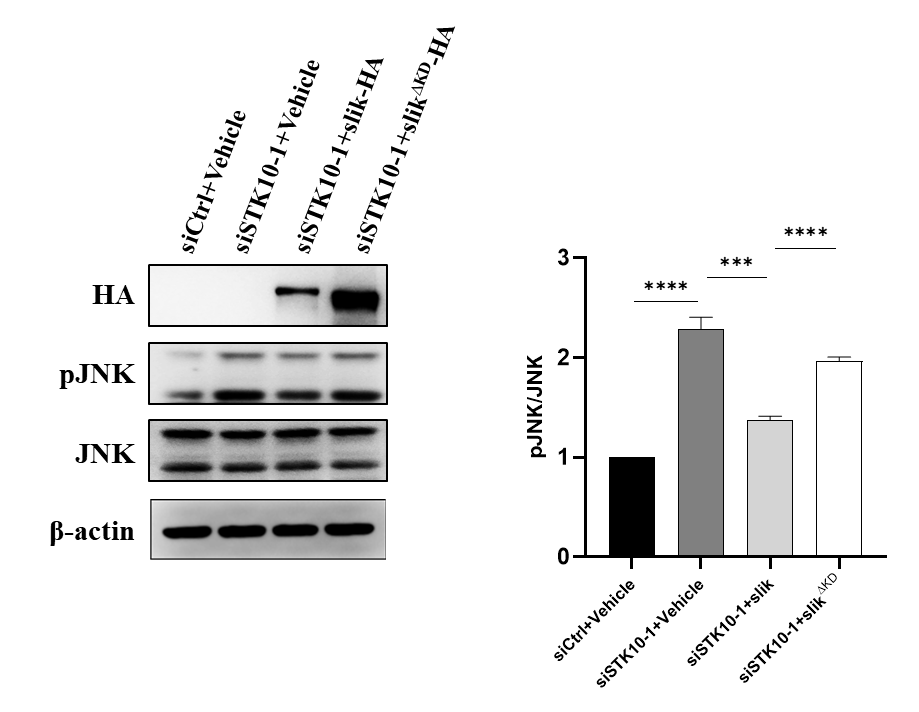
**

**Figure S9.** **Kinase activity is necessary for Slik/STK10 to regulate JNK pathway**

Immunoblot analysis of p-JNK and total JNK protein level in SW480 cells. Compared with the control (lane 1), *STK10* knockdown increased p-JNK level (lane 2), which was effectively suppressed by expressing Slik (lane 3), but not a truncated Slik with kinase domain deletion (lane 4). One-way ANOVA with Bonferroni multiple-comparison test was used to compute P-values, ****p < 0.0001, ***p < 0.001.

**Li et al., Supplement Figure 10**

**
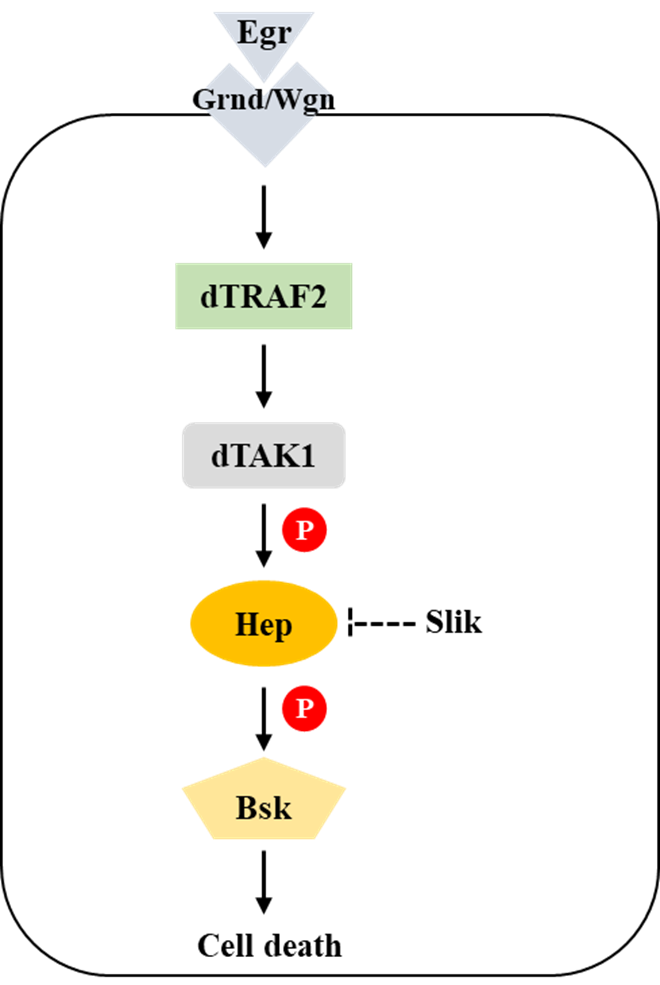
**

**Figure S10.** **Schematic summary of Slik in regulating JNK pathway**

Slik inhibits JNK-mediated cell death. Genetic epistasis analysis suggests that Slik acts upstream of or in parallel to Hep to impede JNK pathway.

**Genotypes in figures:**

**Figure 1**

(A and F) *GMR*-Gal4/+

(B and G) *UAS*-LacZ/*GMR-*Gal4 *UAS*-Hep^CA^

(C and H) *UAS*-Slik/*GMR-*Gal4 *UAS*-Hep^CA^

(D and I) *slik^KG04837^*/+; *GMR-*Gal4 *UAS*-Hep^CA^/+

(K and P) *ptc*-Gal4/+

(L and Q) *ptc*-Gal4 *UAS-*Hep/+

(M and R) *ptc*-Gal4 *UAS-*Hep/+; *UAS*-LacZ/+

(N and S) *ptc*-Gal4 *UAS-*Hep /+; *UAS*-Slik/+

**Figure 2**

(A) *UAS-lacZ-IR*/+; *GMR*‐Gal4/+

(B) *UAS-slik-IR^THU02634^*/+; *GMR*‐Gal4/+

(C) *UAS-slik-IR^BS55626^*/+; *GMR*‐Gal4/+

(D) *GMR*‐Gal4/*UAS-slik-IR^4527R-3^*

(F and K) *ptc*‐Gal4/*UAS-lacZ-IR*

(G and L) *ptc*‐Gal4/*UAS-slik-IR^THU02634^*

(H and M) *ptc*‐Gal4/*UAS-slik-IR^BS55626^*

(I and N) *ptc*‐Gal4/+; *UAS-slik-IR^4527R-3^*/+

(P) From left to right: (1) *UAS-lacZ-IR*/+; *hs*‐Gal4/+ (2) *UAS-slik-IR^THU02634^*/+; *hs*-Gal4/+ (3) *UAS-slik-IR^BS55626^*/+; *hs*-Gal4/+ (4) *hs*‐Gal4/*UAS-slik-IR^4527R-3^*

**Figure 3**

(A) *ptc*-Gal4 *UAS*-GFP *TRE*-RFP/*UAS-lacZ-IR*

(B) *ptc*-Gal4 *UAS*-GFP *TRE*-RFP/*UAS-slik-IR^THU02634^*

(C) *ptc*-Gal4 *UAS*-GFP *TRE*-RFP/*UAS-slik-IR^BS55626^*

(D) *ptc*-Gal4 *UAS*-GFP *TRE*-RFP/+; *UAS-slik-IR^4527R-3^*/+

(E) *ptc*-Gal4 *UAS*-GFP/*UAS-lacZ-IR*

(F) *ptc*-Gal4 *UAS*-GFP/*UAS-slik-IR^THU02634^*

(G) *ptc*-Gal4 *UAS*-GFP/*UAS-slik-IR^BS55626^*

(H) *ptc*-Gal4 *UAS*-GFP/+; *UAS-slik-IR^4527R-3^*/+

**Figure 4**

(A and L) *ptc*‐Gal4/+

(B) *ptc*‐Gal4/+; *UAS-slik-IR^4527R-3^*/+

(C and M) *ptc*‐Gal4/*UAS-lacZ-IR*; *UAS-slik-IR^4527R-3^*/+

(D and N) *ptc*‐Gal4/+; *UAS-slik-IR^4527R-3^*/*UAS-egr-IR*

(E) *ptc*‐Gal4/*UAS-dTRAF2-IR*; *UAS-slik-IR^4527R-3^*/+

(F) *ptc*‐Gal4/+; *UAS-slik-IR^4527R-3^*/*UAS-dTAK1-IR*

(G and O) *ptc*‐Gal4/+; *UAS-slik-IR^4527R-3^*/*UAS-hep-IR*

(H) *ptc*‐Gal4/+; *UAS-slik-IR^4527R-3^*/*UAS-bsk-IR*

(I and P) *ptc*‐Gal4/+; *UAS-slik-IR^4527R-3^*/*UAS-*Bsk^DN^

(J) *ptc*‐Gal4/+; *UAS-slik-IR^4527R-3^*/*UAS-*Puc

**Figure 5**

(A) *hs*-*Flp*/+; *UAS-*GFP/+; *act*>CD2>Gal4 *UAS*-GFP/+

(B) *hs-Flp*/+; *UAS-slik-IR^THU02634^*/+; *act*>CD2>Gal4 *UAS*-GFP/+

(C) *hs*-*Flp*/+; *UAS-slik-IR^THU02634^*/+; *act*>CD2>Gal4 *UAS*-GFP/*UAS-*Bsk^DN^

(E) *en*-Gal4 *UAS*-GFP/*+*

(F) *en*-Gal4 *UAS*-GFP/*UAS-slik-IR^THU02634^*

(G) *en*-Gal4 *UAS*-GFP/*UAS-slik-IR^THU02634^*; *UAS-*Bsk^DN^/*+*

(I) *nub*-Gal4 *UAS*-GFP/*+*

(J) *nub*-Gal4 *UAS*-GFP/*UAS-slik-IR^THU02634^*

(K) *nub*-Gal4 *UAS*-GFP/*UAS-slik-IR^THU02634^*; *UAS-*Bsk^DN^/*+*

**Figure 6**

(A and F) *ptc*‐Gal4/+

(B and G) *ptc*‐Gal4/*+*; *UAS-slik-IR^4527R-3^*/*UAS*-LacZ

(C and H) *ptc*‐Gal4/*+*; *UAS-slik-IR^4527R-3^*/*UAS*-Slik

(D and I) *ptc*‐Gal4/*+*; *UAS-slik-IR^4527R-3^*/*UAS*-STK10

(K) *en*-Gal4 *UAS*-GFP/*+*

(L) *en*-Gal4 *UAS*-GFP/*UAS-slik-IR^THU02634^*

(M) *en*-Gal4 *UAS*-GFP/*UAS-slik-IR^THU02634^*; *UAS*-Slik/*+*

(N) *en*-Gal4 *UAS*-GFP/*UAS-slik-IR^THU02634^*; *UAS*-STK10/*+*

**Figure 7**

(A and E) *GMR*-Gal4/+

(B and F) *UAS*-LacZ/*GMR-*Gal4 *UAS*-Hep^CA^

(C and G) *UAS*-STK10/*GMR-*Gal4 *UAS*-Hep^CA^

(I and M) *ptc*-Gal4/+

(J and N) *ptc*-Gal4 *UAS-*Hep/+; *UAS*-LacZ/+

(K and O) *ptc*-Gal4 *UAS-*Hep/+; *UAS*-STK10/+

**Figure 8**

(A and F) *ptc*-Gal4/*+*

(B and G) *ptc*-Gal4 *UAS-scrib-IR*/*+*; *UAS*-LacZ/+

(C and H) *ptc*-Gal4 *UAS-scrib-IR*/*+*; *UAS*-Slik/+

(D and I) *ptc*-Gal4 *UAS-scrib-IR*/*+*; *UAS*-STK10/+

(K) *ptc*-Gal4 *UAS-scrib-IR*/*+*

(L) *ptc*-Gal4 *UAS-scrib-IR*/*slik^KG04837^*

**Supplement Figure 1**

(B) *UAS*-Egr/+; *GMR-*Gal4/+

(C) *UAS*-Egr/*Df(2R)BSC603*; *GMR-*Gal4/+

(D) *UAS*-Egr/*Df(2R)ED4065*; *GMR-*Gal4/+

(E) *UAS*-Egr/*Df(2R)ED4071*; *GMR-*Gal4/+

(F) *UAS*-Egr/*slik^KG04837^*; *GMR-*Gal4/+

(G) *UAS*-Egr/*UAS-slik-IR^BS55626^*; *GMR-*Gal4/+

**Supplement Figure 2**

(A) *ptc*‐Gal4/+

(B) *ptc*‐Gal4/+; *UAS-slik-IR^4527R-3^*/+

(C) *ptc*‐Gal4/*UAS-lacZ-IR*; *UAS-slik-IR^4527R-3^*/+

(D) *ptc*‐Gal4/+; *UAS-slik-IR^4527R-3^*/*UAS-*P35

**Supplement Figure 3**

(B-B’) *hs*-*Flp*/+; FRT42D *ubi*-GFP/FRT42D

(C-C’) *hs*-*Flp*/+; FRT42D *ubi*-GFP/FRT42D *slik^KG04837^*

**Supplement Figure 4**

(A) From left to right: (1) *hs*‐Gal4/*UAS-*LacZ (2) *hs*‐Gal4/*UAS-*Slik

(B) From left to right: (1) *hs*‐Gal4/*UAS-*LacZ (2) *hs*‐Gal4/*UAS-*STK10

**Supplement Figure 5**

(A-A’’) *hs-Flp*/+; FRT42D *tub*-Gal80/FRT42D; *tub*-Gal4 *UAS*-GFP/+

(B-B’’) *hs-Flp*/+; FRT42D *tub*-Gal80/FRT42D *slik^KG04837^*; *tub*-Gal4 *UAS*-GFP/+

(C-C’’) *hs-Flp*/+; FRT42D *tub*-Gal80/FRT42D *slik^KG04837^*; *tub*-Gal4 *UAS*-GFP/*UAS-*Slik

(D-D’’) *hs-Flp*/+; FRT42D *tub*-Gal80/FRT42D *slik^KG04837^*; *tub*-Gal4 *UAS*-GFP/*UAS-* STK10

(E-E’’) *hs-Flp*/+; FRT42D *tub*-Gal80/FRT42D *slik^KG04837^*; *tub*-Gal4 *UAS*-GFP/*UAS-* Bsk^DN^

**Supplement Figure 6**

(A and F) *GMR*-Gal4/+

(B and G) *UAS*-Egr/+; *UAS*-LacZ/*GMR-*Gal4

(C and H) *UAS*-Egr/+; *UAS*-STK10/*GMR-*Gal4

(D and I) *UAS*-Egr/+; *UAS-hep-IR*/*GMR-*Gal4

**Supplement Figure 7**

(A and E) *ptc*-Gal4 *UAS-scrib-IR*/*+*; *UAS*-LacZ/+

(B and F) *ptc*-Gal4 *UAS-scrib-IR*/*+*; *UAS*-Bsk^DN^/+

(C and G) *ptc*-Gal4 *UAS-scrib-IR*/*+*; *UAS*-Puc/+
